# Effect of the One-Child Policy on Influenza Transmission in China: A Stochastic Transmission Model

**DOI:** 10.1371/journal.pone.0084961

**Published:** 2014-02-06

**Authors:** Fengchen Liu, Wayne T. A. Enanoria, Kathryn J. Ray, Megan P. Coffee, Aubree Gordon, Tomás J. Aragón, Guowei Yu, Benjamin J. Cowling, Travis C. Porco

**Affiliations:** 1 F.I. Proctor Foundation, University of California San Francisco, San Francisco, California, United States of America; 2 Center for Infectious Diseases and Emergency Readiness, School of Public Health, University of California, Berkeley, California, United States of America; 3 Division of Epidemiology, School of Public Health, University of California, Berkeley, California, United States of America; 4 West of China Institute of Environmental Health, Northwest University for Nationalities, Lanzhou, Gansu, China; 5 School of Public Health, The University of Hong Kong, Hong Kong, China; 6 Department of Epidemiology & Biostatistics, University of California San Francisco, San Francisco, California, United States of America; National Institutes of Health, United States of America

## Abstract

**Background:**

China's one-child-per-couple policy, introduced in 1979, led to profound demographic changes for nearly a quarter of the world's population. Several decades later, the consequences include decreased fertility rates, population aging, decreased household sizes, changes in family structure, and imbalanced sex ratios. The epidemiology of communicable diseases may have been affected by these changes since the transmission dynamics of infectious diseases depend on demographic characteristics of the population. Of particular interest is influenza because China and Southeast Asia lie at the center of a global transmission network of influenza. Moreover, changes in household structure may affect influenza transmission. Is it possible that the pronounced demographic changes that have occurred in China have affected influenza transmission?

**Methods and Findings:**

To address this question, we developed a continuous-time, stochastic, individual-based simulation model for influenza transmission. With this model, we simulated 30 years of influenza transmission and compared influenza transmission rates in populations with and without the one-child policy control. We found that the average annual attack rate is reduced by 6.08% (SD 2.21%) in the presence of the one-child policy compared to a population in which no demographic changes occurred. There was no discernible difference in the secondary attack rate, −0.15% (SD 1.85%), between the populations with and without a one-child policy. We also forecasted influenza transmission over a ten-year time period in a population with a two-child policy under a hypothesis that a two-child-per-couple policy will be carried out in 2015, and found a negligible difference in the average annual attack rate compared to the population with the one-child policy.

**Conclusions:**

This study found that the average annual attack rate is slightly lowered in a population with a one-child policy, which may have resulted from a decrease in household size and the proportion of children in the population.

## Introduction

The one-child-per-couple policy in China was introduced in 1979 in an effort to raise living standards by slowing population growth. Subsequently, the policy reduced fertility rates [1], [2] and household sizes, with only one dependent child found in most households. The total birth rate dropped from 2.90, before the policy was introduced, to 1.94 among women over 35 years of age, and to 1.73 among women under 35 years old in 2001. Women's preferences for smaller families have changed (35% prefer one child and 57% prefer two children according to a study in 2001) [3]. The total fertility rate decreased from 2.9 in 1979 to 1.7 in 2004, with a rate of 1.3 in urban areas and less than 2.0 in rural areas. This trend has created a distinct demographic pattern for nearly a quarter of the world's population, resulting in Chinese urban families with predominantly one child and rural families with predominantly two children [4].

The spread of infectious diseases may depend on demographic characteristics, environmental changes, consumption behaviors (eating, drinking, culinary culture, etc.), other behaviors (sexual contacts, drug use, hospital procedures, etc.), and host conditions (malnutrition, diabetes, immune status, etc.) [5]. While the one child policy has had economic, demographic, and sociological ramifications far beyond the scope of infectious disease transmission, it is important to understand the consequences for influenza dynamics, in part because China and Southeast Asia lie at the center of a global transmission network of influenza [6]. Demographic changes may affect influenza transmission dynamics because children have an increased susceptibility due to lower immunity. Moreover, increased viral shedding and longer infectious periods in children lead to more influenza among susceptible populations [7]. Demographic characteristics have been incorporated into many modeling studies [8], [9], [10] to help understand the effects on transmission of influenza or the socioeconomic impact of mitigation strategies [11], [12]. Household composition is an important determinant of the transmission of respiratory pathogens including influenza [13], [14], [15], [16], [17], [18], [19] and remains an important feature of recent transmission models [20], [21], [22], [23], [24], [25].

This paper presents a study focusing on the indirect effects of demographic changes on influenza transmission. We developed a continuous-time, event-driven, individual-based stochastic simulation model for influenza transmission in a dynamic population. We used this model to simulate transmission while assuming different demographic control policies: the one-child policy, the absence of any control policy, and a strict one-child policy. The strict one-child policy was introduced to compare influenza transmission rates with a hypothetical one-child policy to rates with an actual one-child policy, since two or more children are often allowed in rural areas and for ethnic minorities [4]; the existing census data do not reflect the effects of truly restricting families to one child. The model was used to simulate 30 years of influenza transmission in a dynamic population as follows: (1) we initialized the population using 1975 demographic data (four years before a one-child policy was fully launched in China); (2) we calibrated the population projections by fitting the simulated population with the one-child policy to the census and compared the simulated population without the one-child policy with projections from previous literature [26], [27] in which population growth was predicted under different demographic control policies; (3) we calibrated the influenza-specific parameters by fitting the annual attack rate and secondary attack rate from the reported literature [7], [28], [29], [30], [31], [32], [33]; and (4) we compared the simulated annual attack rate and secondary attack rate in simulated populations with and without the one-child policy. In the plenary sessions of the 2011 Chinese People's Political Consultative Conference and the National People's Congress, a two-child policy was proposed to start as early as 2015 [34]. Experts suggested that the one-child policy may threaten China's economic growth due to the increase in the number of older people, a decrease in the number of younger workers, as well as a sex-ratio imbalance [34]. Because a two-child policy was proposed to start as early as 2015 [34], we also forecasted influenza transmission over a ten-year period (2015 to 2024) in a population with a two-child policy.

## Methods

Our model has three main features: (1) influenza transmission, (2) population demographics, and (3) dynamic network structure. We used a susceptible-exposed-infectious-recovered (SEIR) model which included waning immunity and seasonality of influenza transmission. We used census data [35], [36], [37], [38], [39] (Table 1) from China, to construct a population with demographic changes under a one-child policy. A simple dynamic network structure was used to group people with household links, school links and social links, allowing influenza to be transmitted along these links in the network while changing the state of each individual (S, E, I and R). The model structure is described in the section titled *Model Structure* (and in the Text S1 and Figure S1). Influenza transmission parameters were calibrated using Approximate Bayesian Computation (ABC) [40], [41] as described in the *Calibration* section (we chose parameters' ranges based on both the English and Chinese literature [42]). The *Computation* section briefly discusses the implementation and computations based on calibrated parameters (Table 2); a more detailed description can be found in the Text S1.

**Table 1:** Demographic parameters.

Descripton	Values	Age-specific	Symbol	Distribution
Population Age	0–100	Yes	*age_distr_*	Age distribution from census [26], [27], [35], [36], [37], [38]
Household size	1–10	No	*h_distr_*	Household size distribution, in census [26], [27], [35], [36], [37], [38]
Household age structure	0–100	Yes	*hs_age_*	Distribution by generation in one household, from census [26], [27], [35], [36], [37], [38]
Mortality probability	0–0.33	Yes	*sv*	Mortality rate from 1975 to 2009 [26], [27], [35], [36], [37], [38]
Fertility probability	0–0.30	Yes	*mf*	Fertility rate of female from 1975 to 2009 [26], [27], [35], [36], [37], [38]
Sex ratio	0.48–0.52	Yes	*sex_distr_*	Sex ratio from 1975 to 2009 [26], [27], [35], [36], [37], [38]
Age to leave from home as a single household	15–18	Yes	*age_split_*	Uniform
Time of leaving home in a specific year	1–365	-	*t_split_*	Uniform
One-child policy: apply dynamic fertility rate	Bool	Yes	*policy_1_*	Fertility rate of females from 1975 to 2009 [26], [27], [35], [36], [37], [38]
No one-child policy: apply static fertility rate	Bool	Yes	*policy_2_*	Fertility rate of females in 1975 [26], [27]
Strict one-child policy: apply dynamic fertility rate, and in the condition that a female cannot give more birth if she already has a child	Bool	Yes	*policy_3_*	Fertility rate of females from 1975 to 2009
Two-child policy: increase the fertility rate for the female who has no child yet, use the static fertility rate in 2009 as that in the years from 2015 to 2024	Bool	Yes	*policy_4_*	Fertility rate of female in 2009, multiplying by 2.

**Table 2:** Influenza transmission parameters.

Interpretation	Initial Value	Lower	Upper	Symbol	Reference
Latent period, day	1	0.5	3	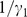 , 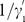	[43], [45], [50], [84]
Asymptomatic infectious period, day	1	0.5	2	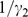 , 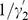	[22], [50], [85]
Symptomatic infectious period, day	2	1	5	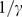	[43], [44], [45], [50], [51], [52]
Probability of non-mild influenza	0.75	0.5	0.8	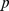	[46], [47], [49]
Immunity, after recovery	1	0.8	1	-	[31]
Contact rate per day outside household	16	-	-	*Contact_casual_*	*NOTE*: *the transmission and contact parameters are not precisely characterized; we chose values leading to an approximate R_0_ about 1.5 in the first influenza season, and conducted exploratory analysis to estimate the effect of the one-child policy over a very wide range of possible values for these parameters.*
Contact rate per day between two household members	10	-	-	*Contact_house_*	
Contact rate per day in school	10	-	-	*Contact_school_*	
Base transmission probability per contact	0.08	0.0008	0.03	*P_base_*	
Immunity loss per year, fraction	0.1	0.001	0.2	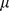	[31]

### Model Structure

#### Natural history of influenza

Individuals infected with the influenza virus first pass through a latent period when they are asymptomatic and not infectious. We assumed that viral shedding does not take place during the latent period, and that the mean duration of the latent period is 1 to 2 days [43], [44], [45]. For influenza, the infectious period is assumed to begin about one day before the symptomatic period [44]. In general, individuals infected with influenza may be asymptomatic, and yet still shed the virus. The proportion of transmission by asymptomatic individuals is assumed to be one-third to one-half that of influenza-infected symptomatic individuals [46], [47], [48], [49]. The mean period during which a person may be asymptomatic but infectious is assumed to be 1 day [50]. Individuals are assumed to become symptomatic and infectious with an average duration of 1.5 to 3.8 days [43], [44], [45], [51], [52].

**Figure 1:**
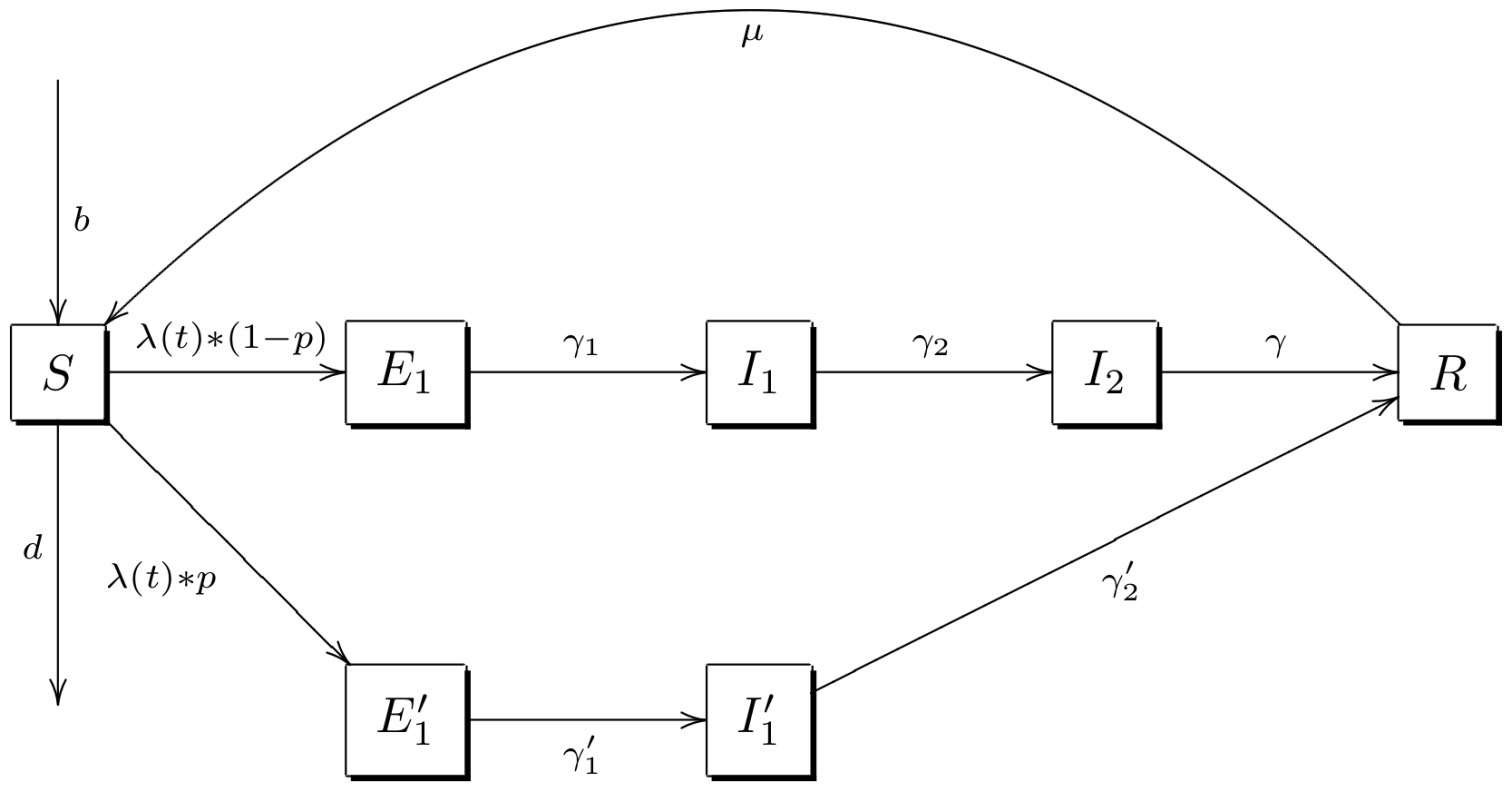
Progression of the model. Given a time *t*, each individual in the model is in one state of 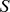 (susceptible), 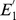 (mild exposure), 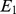 (not mild exposure), 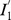 (mild asymptomatic infectiousness), 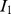 (not mild asymptomatic infectiousness), 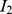 (symptomatic infectiousness) and 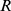 (recovered with immunity), and the population's inflow and outflow are represented by each individual's age-specific death rate *d* and age-specific fertility rate *b*.

Mathematically, we represent the course of influenza according to the diagram shown in Figure 1 (and Figure S2). In this model, we classified influenza as being mild or not being mild; individuals in each severity type progress to different stages. Mild cases and non-mild cases are classified as infected prior to all symptoms and infectiousness (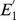 and 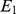), infectious but asymptomatic (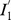 and 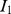), or recovered with strain specific immunity (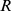). The non-mild cases may be symptomatic and infectious as well (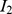), which occurs after asymptomatic infectiousness (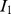). Table 2 lists the durations between stages. A recovered individual loses immunity with rate *μ*, reverting to the uninfected susceptible (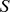) stage. We assume that individuals have age-specific death rate *d*, and a birth rate *b*; these dynamic population demographic features which are represented by the death and birth of each individual, will be described in the demographic description section given below. For a specific individual, we assume that the duration time between two stages is randomly chosen from an exponential distribution with a given rate.

#### Immune Escape and Seasonality

To model antigenic drift, our model is designed such that every individual has a maximum immunity level immediately following recovery from infection by a particular strain of influenza, but this immunity gradually wanes to zero over 3 to 8 years [28], [50]. Following reinfection, the immunity level is restored to the maximum value and declines at the same rate thereafter.

The underlying causes of influenza seasonality remain unclear [53], [54], [55], despite many studies postulating possible causes. Suggested causes have included changes in human mixing patterns or fluctuations in human immunity and environmental humidity [56]. The transmission of seasonal influenza tends to increase substantially from November to February in the Northern hemisphere and from May to August in the Southern hemisphere [57]. To incorporate seasonality of influenza transmission into our model, we modeled the transmission probability per contact as a sinusoidal function of time [57] according to 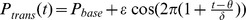, so that the transmission probability, *P_trans_*, varies during the course of the epidemic. Here, *P_base_* is the baseline transmission probability, 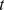 is time, and *ε* (where we assume *−P_base_*<*ε*<*P_base_*) characterizes the degree of seasonality (*ε* = 0 corresponds to no seasonal variation at all). We let *δ* denote the total duration of an epidemic season (for instance, 365 days in this model) and 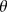 (an offset from time 0) is the peak time of an epidemic season. Our model adopted 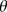 as November 15, corresponding to northern China where influenza peaks in the winter [58]. In this model, the probability of infection for each individual depends on the immunity level, seasonality, and the contact rates (please see the Text S1 for more details).

#### China's Demographic Data

The demographic data were taken from the Population Statistics Yearbooks for China, and from five censuses carried out in 1952, 1964, 1982, 1990 and 2000 [35], [36], [37], [38], [39]. Some demographic data sources were extracted from previous articles [26], [27], [39], [59], [60] in which the population growth under different population control policies were predicted. Key demographic parameters used in our simulation included age, household size, age-specific death rates, and age-specific fertility rates, as shown in Table 1. Initially, we stochastically sampled age and household size from distributions fitted to the demographic data [26], [39], [60]. We used dynamic age-specific fertility rates and death rates from year 1975 to 2009 to simulate the population growth under conditions of the one-child policy; calibration details of the age-specific fertility rates can be found in Text. Population projections without the control of a one-child policy were implemented by assuming a static age-specific fertility rate (from 1975) and fixing the birth rate to the same value that it was in 1975 (which, of course, corresponds to an unrealistic population trajectory). We also analyzed the assumption of a very strict one-child policy that allows one female to have only one child in her life—this is stricter than the one-child policy as actually implemented. Finally, we conducted a simple projection of the population with a proposed two-child policy (from 2015 to 2024), which allows one female to have two children. It was implemented by increasing the fertility rate for nulliparous females. To calibrate the population, we fit the age-specific population number of each year and the average household size of each year to the census data, then compared the population projections of our model with the census data and projections described in other studies [26], [39], [60].

#### Dynamic Network Structure

**Figure 2:**
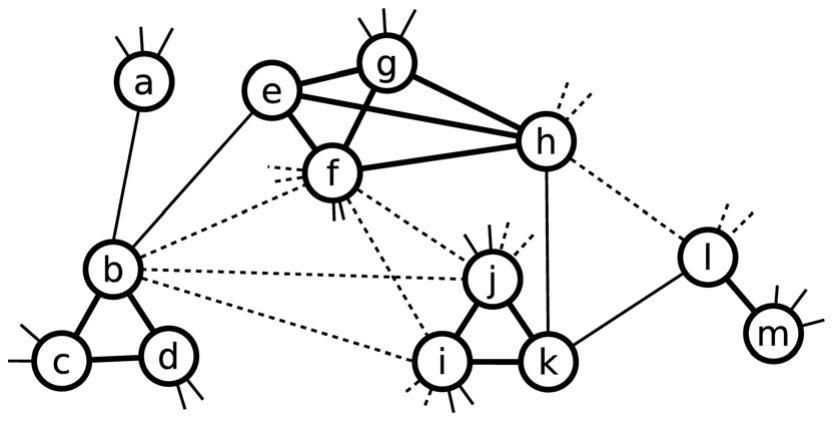
Dynamic Network Structure. The population contact network of this model consists of every individual's household, school (if in school age) and casual links. This small part of the network has 13 individuals in 5 households with different sizes: individual *a* is in a 1-member household, individuals *l* and *m* are in a 2-member household, individuals *b*, *c* and *d*, and individuals *i*, *j* and *k* are in two 3-member households, individuals *e*, *f*, *g* and *h* are in a 4-member household. Individuals in each household are linked each other by thick lines. Each individual has some casual links (linked by thin lines) to other non-household members. School age individuals *b*, *f*, *i*, *j*, *h* and *l* are in two different schools and linked by dotted lines (the schoolmate relationship). Individual *b* has two household members (*c* and *d*), two visible casual contacts (*a* and *e*), and three visible schoolmates (*f*, *i* and *j*), other social contacts and schoolmates of *b* are not shown in this small part of contact network. If *b* was an index case, the household contacts would be at highest risk of being infected due to the higher contact rates among household members than the casual and school contacts (for the contact rates of different link types, please see Table 2).

We simulated the transmission of influenza using a simple dynamic network structure shown in Figure 2. Specifically, we assumed that each individual is located in a household and links to other household members, and we assumed that each individual has several links to other individuals outside of his/her household. These links outside the household represent contacts in the community and an individual has a lower relative contact rate with outside links than with household links. For school-aged individuals, we assume that they are in primary and middle schools, and have school links to all of their schoolmates. The contact network of this model consists of each individual's household contacts, school contacts and casual contacts, and its dynamic is reflected by updating each individual's household, school and casual contacts which will be discussed in turn.

#### Household Contacts

Each individual in the model has household links that are initialized by grouping individuals into households based on the household size distribution data of China in 1975, and linking all household members of each household. During simulation, each individual's household links are updated dynamically (1) when the individual leaves his/her household between his/her age 14 and 18 years as a household with one-member, (2) when the single individual over 18 years of age has found (with a partnership searching rate per year) another single over 18 years of age to live with as a two-member household, (3) at the time the individual dies (with a dynamic age-dependent mortality rate), or (4) at the time the individual or one of the other family members gives birth to a baby (with a dynamic age-dependent fertility rate). The dynamic age-dependent mortality rate and the dynamic age-dependent fertility rate are from the population data of China from 1975 to 2009. During the simulation, an individual's mortality rate and fertility rate depend on the current simulated year and the individual's current age. The partnership searching rate per year is calibrated to fit to the observed household size from 1975 to 2009. The dynamic age-dependent fertility rates under the other three scenarios are assumed to be zero if the individual already has more than one child for the strict-one-child-policy, the same as the fertility rates in 1975 for the absence of one-child policy, and doubled from 2015 to 2024 for the two-child-policy.

#### School Contacts

Each individual whose age is between the primary-school-age of 6 and 12 years or between the middle-school-age of 13 and 18 years has school links that are initialized using the primary and middle schools' statistical data of Gansu province in China in 1975, and are updated annually by reassigning all individuals with school ages into primary or middle schools according to year-dependent average school size from 1976 to 2009, or are updated at the time the individual dies with the dynamic age-dependent mortality rate.

#### Casual Contacts

Each individual may have several random contacts per day with a daily contact rate *contact_casual_*  = 16. Once an individual becomes infectious, all of his/her casual contacts during the infectious period are randomly chosen from the population and their contacting times are predicted and scheduled using an exponential distribution with the casual contact rate per day, *contact_casual_*.

#### Transmission via the Network

Once an individual becomes infectious, an infectious period will be generated using an exponential distribution with recovery rate. During the individual's infectious period, the contact times between he/she and each of his/her household members are stochastically scheduled using an exponential distribution with the contact rate per household member per day, *contact_house_* = 10; transmission between the infectious individual and the susceptible household contacts will take place at the scheduled contact times. Similarly, the casual contacts of the infectious individual during the infectious period are randomly chosen from the entire population, and the contact times between the infectious individual and his/her casual contacts are scheduled using an exponential distribution with a casual contact rate per day, *contact_casual_* = 16. Transmission between the infectious individuals and the susceptible casual contacts will be active at the scheduled times. In addition, the contacts between the infectious individual and his/her schoolmates during his/her infectious period are randomly picked from the individual's school links and are scheduled by an exponential distribution with school contact rate per day, *contact_school_* = 10. The transmission between the infectious individual and the susceptible school contacts will be active at the scheduled times. Once a scheduled transmissible contact takes place between the infectious individual and one of his/her susceptible household members, schoolmates, or casual contacts, a successful transmission will be completed with a transmission opportunity which is a product of the seasonal transmission probability per contact, *P_trans_*, and the chance of immune escape, 1 - *M_i_*(*t*), where *M_i_*(*t*) (defined in the Text S1) is a dynamic immunity level of a susceptible individual *i* at time *t*. The dynamic immunity level of an individual depends on his/her infection history, the immunity waning rate per year and the current time.

### Model Assumptions

The model is initialized with 10,000 individuals whose ages are generated from the age distribution of China in 1975. The household links for each individual are initialized with household size distribution of China in 1975, and the school links for each school age individual are initialized with the average school size of Gansu province in China in 1975. Casual contacts of each individual are randomly selected from the population with a casual contact rate per day *contact_casual_* = 16. Five exogenous infectious cases with the same influenza strain are introduced into the population on November 15th in 1975 to start influenza transmissions via the contact networks of all individuals. At the beginning of the simulation, we assume that all individuals are completely susceptible. Once an individual recovered from an infection, he/she will have a 100% immunity level which wanes with 10% immunity loss rate per year (*μ* = 0.1). The demography-dependent dynamic network of the population is reflected by updating household links and school links of each individual as stated above, which also depends on the scenario of population control policy for the current simulation. As a base scenario, we assume that the one-child policy is active, thus the mortality rates for ages 0 to 120 years and the fertility rates for ages 16 to 50 years of the population are updated each year with the census data from 1976 to 2009. Similarly, the average sizes of primary and middle schools are updated each year with the observed school data of Gansu province in China from 1976 to 2009. Another five exogenous cases with the same strain are introduced into the population on November 15th in each year from 1976 to 2009, and we assume that there are no changes in the influenza natural history parameters during the course of over 30 years. The first influenza season in 1975 is used to calibrate the household, school and casual contact rates as well as the base transmission probability per contact by choosing their values leading to an approximate *R*_0_ around 1.5. The first four-year influenza seasons from 1975 to 1979 are used to enable the totally susceptible population in 1975 to have partial immunity in 1979. The following influenza seasons from 1979 to 2009 are used to calibrate influenza natural history parameters and transmission parameters by fitting the simulated average annual attack rate and average secondary attack rate to the observed data.

### Calibration

Existing census data reflect those demographic changes caused by the one-child policy as actually implemented. In order to assess what would have occurred in the absence of such a policy or other demographic changes, we assumed a static fertility rate of that in 1975 for females. However, a strict one-child policy includes the assumption that there is no chance for a female who already has a child to give birth to a second child, an assumption that does not hold in practice. To calibrate the demographic component of our model, we first fitted the population projection with the available demographic data, as well as with other population projections [26], [27] in which they predicted population with a one-child policy and other control measures (see Figure S3(A)). Then, we fitted the population age distribution of each year to demographic data in the years from 1975 to 2004, (see Figure S3 (B)). Finally, we required that the average household size (see Figure S3(C)) corresponded to the census data in 1964, 1982, 1990 and 2000, which reported average household sizes of 4.43, 4.42, 3.96 and 3.44 in these years, respectively [35].

We calibrated the model using eight influenza transmission parameters: (1) mean duration of the latent period, (2) mean duration of the asymptomatic infectious period, (3) mean duration of the symptomatic infectious period, (4) probability that a case will be mild, (5) immunity waning rate, (6) the degree of immunity following infection, (7) transmission probability per contact, and (8) contact rate between two household members (Table 2). Parameters (1), (2), (3) and (4) are age-dependent parameters with 5 age categories: 0 to 4, 5 to 9, 10 to 25, 26 to 49, and 50+ years. To calibrate these parameters, we chose parameter sets randomly from a uniform distribution with given upper and lower bounds (assuming independence among parameters). The annual attack rate (averaged over 30 years) and the simulated household secondary attack rate (averaged over 30 years, and the rate of each year was averaged over all households with index cases) were computed from each set of parameters. Simulations yielding average annual attack rate (*AR*) within the range (0.1, 0.2)[28], [29], [30], [31], and secondary attack rate (*SAR*) inside the range (0.09,0.32)[7], [32], [33], [61], [62], [63], were considered plausible; calibration was done by Approximate Bayesian Computation [40], [41]. For details of the *AR* and *SAR* we cited, please see Tables S1 and S2. For each household with an index case, we calculated the secondary attack rate based on the proportion of household contacts who were infected by the index case in the household during the infectious period of the index case [62], [64]. The *SAR* was averaged by using the secondary attack rates of all households with index cases. This calculation of the *SAR* includes partially immune household contacts [7], [32], [33], [61], [62], [63]. Simulations were run for 4000 sets of parameters, resulting in 646 parameter sets that fit the acceptable *AR* and *SAR* ranges stated above. Parameter sets having higher or lower values of *AR* or *SAR* were excluded. Finally, we used the 646 fitted (non-excluded) parameter sets and used them in the model to predict and study influenza transmission in the population under three scenarios: the one-child policy, the absence of a one-child policy, and the strict one-child policy.

### Computation

The individual-based model was implemented and programmed in C++ [65] and R [66] following our previously published agent-based transmission models [67], [68]. C++ was used for the main simulation program and R for the analysis of data generated by the main simulation program. To add scalability for simulations of large population sizes, we used an agent-based platform *ABM*++ [69] which supports parallel and cluster computing. Simulations were performed on the *RTI MIDAS* cluster, a cluster with 36 compute nodes with a total of 400 compute cores and 786 GB of distributed memory, running Linux distribution of *CentOS* v5.5. The running time for a single run of the model varied with input parameters in Tables 1 and 2. Given a fitted set of parameters with the one-child policy and an initial population size of 10000, it took about 500 to 800 seconds for a single run on one compute core with a speed of 2.30 GHz in the cluster.

## Simulations

We simulated 30 years of influenza transmission in a representative population of initial size 10000 under three different scenarios: a population with a one-child policy (), a population without a one-child policy (), and a population with a strict one-child policy (), (following “one-child policy” represents ). Under each of the scenarios, we used 646 fitted sets of parameters (described in the *Calibration* section) to simulate influenza transmission. Each scenario was simulated 100 times and the annual and secondary attack rates were averaged among 100 simulated *AR*s and *SAR*s. We then computed the partial rank correlation coefficients (*PRCC*) [67], [70] for each input parameter and the annual attack rate under the three different policy scenarios using the 646 sets. When the *PRCC* is close to zero, the value of the parameter has little relation to the simulation output (see the Text S1). The *PRCC* values of key parameters are listed in Table 3. Finally, we calculated the annual and secondary attack rates experienced by the population under the three policy scenarios.

**Table 3:** Sensitivity analysis for the annual attack rate *PRCC*: Partial Rank Correlation Coefficient.

*PRCC* with	Δ*AR* of *policy*_2_ and *policy*_1_	*AR policy*_2_	*AR policy*_3_	*AR policy*_1_
Transmission probability	0.493	0.777	0.843	0.740
Immunity waning rate	0.477	0.668	0.658	0.593
Immunity after infection	−0.138	−0.133	−0.068	−0.085
Latent period	0.143	0.180	0.212	0.131
Asymptomatic infectious period	0.150	0.261	0.320	0.227
Symptomatic infectious period	0.043	0.189	0.222	0.191
Not Mild case probability	0.167	0.135	0.007	0.034

Note: *policy*_1_ is absence of intervention, *policy*_2_ is one-child policy and *policy*_3_ is strict one-child policy.

## Results

**Figure 3:**
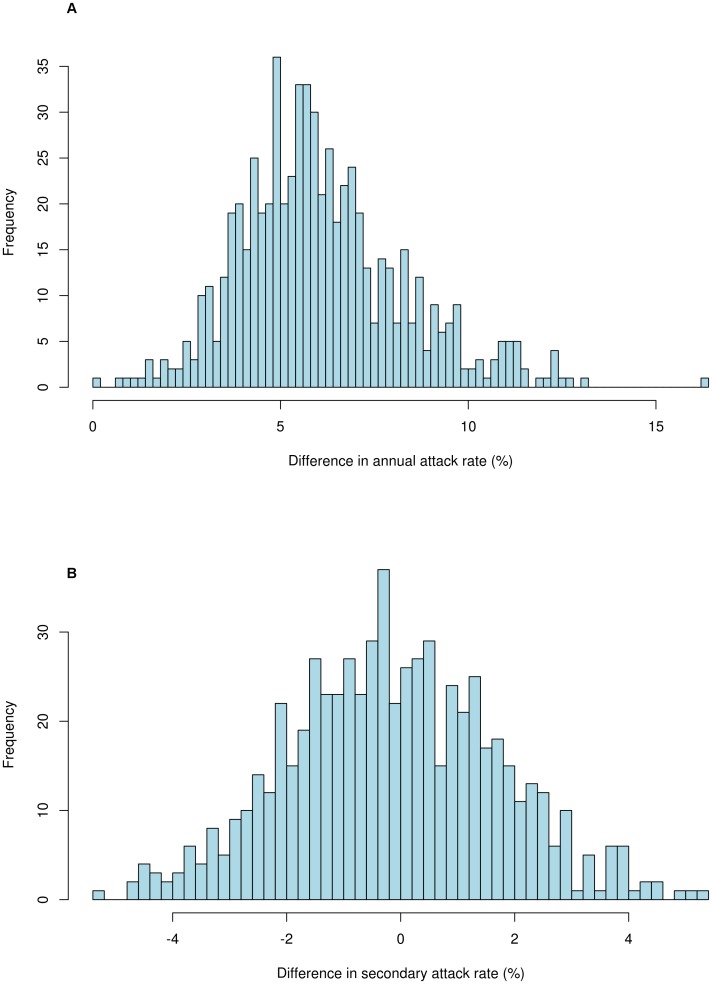
*AR* and *SAR* differences between populations without the one-child policy and with the one-child policy. (A) Average difference in annual attack rate (Δ*AR:* 6.08% (SD 2.21%)) between populations without the one-child policy and with the one-child policy, based on 646 calibrated parameter sets which yielded the annual attack rates between 10% and 20%, and secondary attack rates between 9% and 32%. For each parameter set, we simulated the influenza trajectories under two demographic control policies, and then computed the difference in average annual attack rates over 30 years between two policies. (B) Difference in secondary attack rates (Δ*SAR*: −0.15% (SD 1.85%)) between populations without one-child policy and with the child-policy, based on 646 calibrated parameter sets which yielded the annual attack rates between 10% and 20%, and the secondary attack rates between 9% and 32%. For each parameter set, we simulated the influenza trajectories under two demographic control policies, and then computed the difference in average secondary attack rates over 30 years between two policies.

To explore the influenza transmission factors that are likely affected by the one-child policy, we estimated the average differences in the annual attack rate (Δ*AR*) and the secondary attack rates (Δ*SAR*) in the populations without and with the one-child policy control. We found that the population without the one-child policy had an average annual attack rate that was slightly higher than the population with the one-child policy. The distribution of the difference of annual attack rates with a mean of 6.08% per year (with standard deviation (SD) 2.21%) using 646 fitted sets of parameters, in Figure 3(A), shows that all the values reflecting the Δ*AR*s between population without one-child policy and population with one-child policy are positive for all sets of parameters. Here, each value of Δ*AR* is the difference of the average annual attack rates over 30 years between two different policies. This supports the notion that the one-child policy gradually reduced the annual attack rate. The decrease in annual attack rates may be caused by the smaller household sizes and the decreased proportion of children in the population resulting from the one-child policy. The distribution of Δ*SAR*, in Figure 3(B), shows that the expectation of the Δ*SAR* is −0.15% per household per year (SD 1.85%) and there is no significant difference of secondary attack rates with the one-child policy introduced. However, the one-child policy had little to no discernible effect on the secondary attack rates. A larger population size gave similar results as stated above.

**Figure 4:**
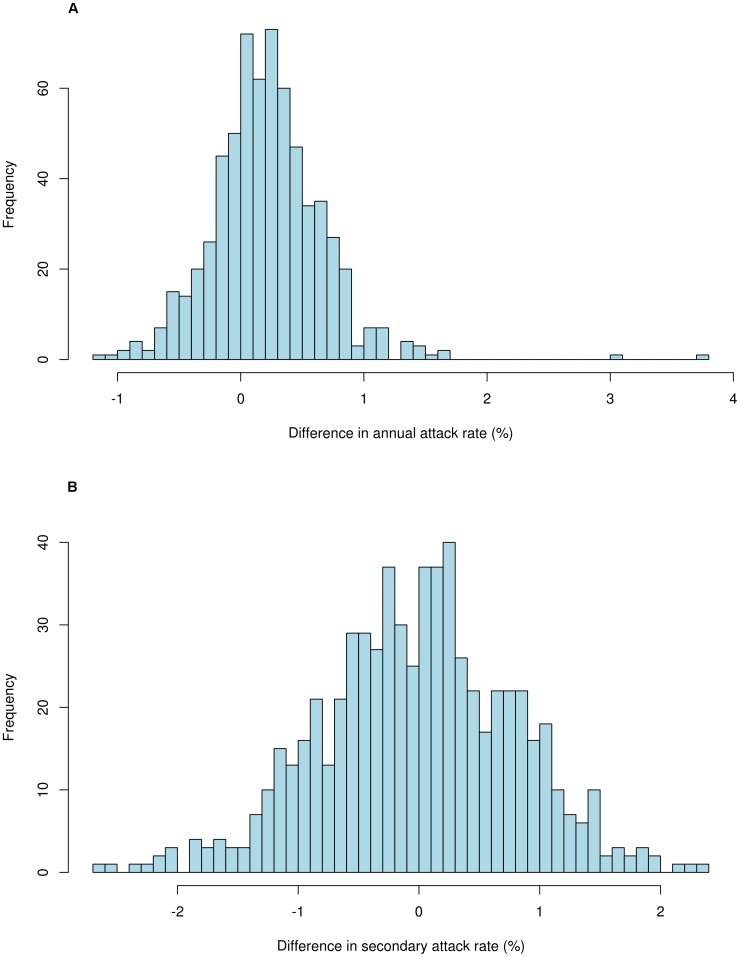
*AR* and *SAR* differences between one-child policy and two-child policy (10 years: 2015 to 2024). (A) Δ*AR* (0.22% (SD 0.46%)) between one-child and two-child policies based on 646 calibrated parameter sets which yielded the annual attack rates between 10% and 20% and the secondary attack rates between 9% and 32%. For each parameter set, we simulated the influenza trajectories under two demographic control policies, and then computed the difference in average annual attack rates over 10 years (2015 to 2024) between two policies. (B) Δ*SAR* (−0.02% (SD 0.81%)) between one-child and two-child policies based on 646 calibrated parameter sets which yielded the annual attack rates between 10% and 20% and the secondary attack rates between 9% and 32%. For each parameter set, we simulated the influenza trajectories under two demographic control policies, and then computed the difference in average secondary attack rates over 10 years (2015 to 2024) between two policies.

We performed the same comparisons of the Δ*AR* and Δ*SAR*, comparing populations with the existing one-child policy with a hypothetical two-child policy. We assumed the two-child policy from 2015 to 2024; the simulations for a 10-year transmission period (Figure 4) did not show significant differences of Δ*AR* and Δ*SAR* (0.22% per year (SD 0.46%) and −0.02% per household per year (SD 0.81%), respectively).

**Figure 5:**
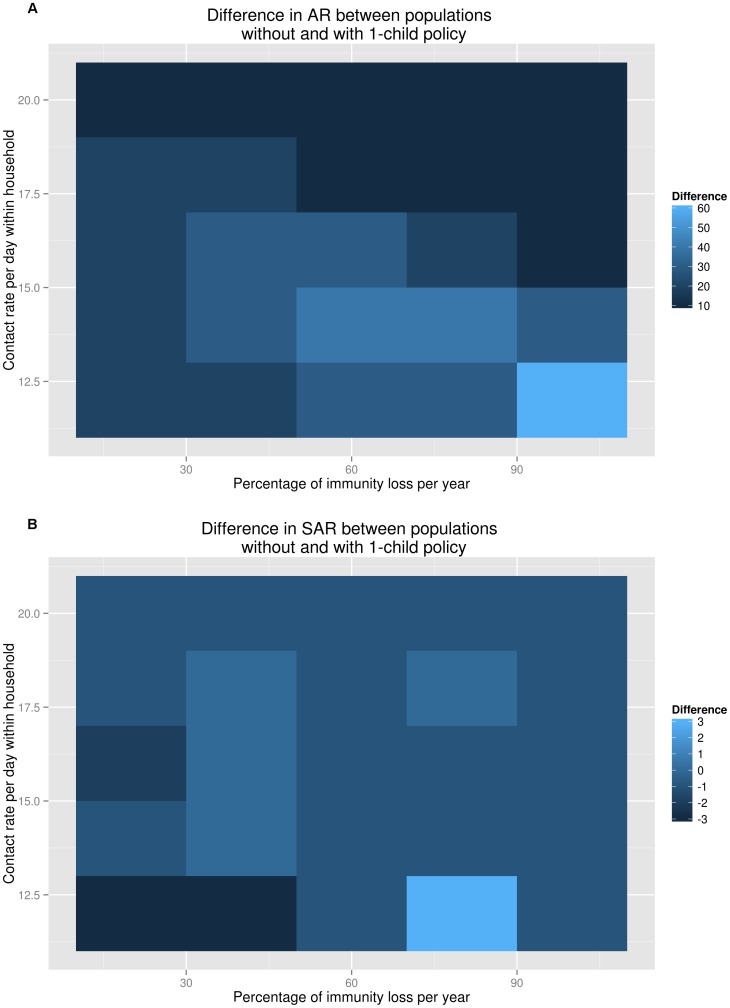
*AR* and *SAR* differences under assumptions of different contact and immunity loss rates. (A) Varying the value of contact rate per day between any two members in a household (from 12 to 20) and the value of immunity loss rate per year (from 20% to 100%) yielded that under the scenario of 12 of household contact rate and 100% of immunity loss per year, the *AR* in the population without the one-child policy could be 60% higher than the *AR* in the population with the one-child policy. (B) By varying the values of contact rate per day between any two members in a household (from 12 to 20) and the immunity loss rate per year (from 20% to 100%), the *SAR* in the population without one-child policy could be 3% higher than the *SAR* in the population without the one-child policy, when the contact rate per day in household is 12 and the immunity loss rate per year is 80%.

In addition, we conducted sensitivity analyses by increasing the contact rate per day within household and the immunity loss rate per year and varying their values from 12 to 20 for the contact rate and from 20% to 100% for the immunity loss rate in order to compare the difference in *AR* and the difference in *SAR* between populations without and with the one-child policy (Figures 5 (A) and (B)). Changes in household structure and the proportion of children in the population as a result of the one-child policy could have more effects on the *AR*, and the difference in *AR* could be as high as 60% under a scenario of very high immunity loss rate per year (Figures 5(A)). However, the results showed that the difference in *SAR* was not very sensitive to the contact rate in the household and the immunity loss rate (Figures 5(B)).

## Discussion

The one-child policy has been applied in China for over 30 years, causing great changes in the demographic composition of the Chinese population. To address the impact of demographic changes caused by the one-child policy (or similar changes which may have arisen for other reasons) on influenza transmission, we developed a continuous-time individual-based, stochastic, simulation model for influenza transmission in dynamic populations with the support of available demographic data. After calibrating the simulated population with available demographic data and published attack rates, we simulated 30 years of influenza transmission under three assumptions: a population with a one-child policy, a population without a one-child policy, and a population with a strict one-child policy. This study provides some evidence that demographic changes caused by demographic policy may slightly affect influenza transmission in populations. Simulated results from this model show that populations without child-bearing policies have slightly higher annual attack rates than populations with a one-child policy. We did not find significant differences in the secondary attack rates between populations with a one-child policy and populations without it. We predicted influenza transmission over 10 years (2015 to 2024) in a population with a hypothetical two-child policy, and found negligible differences of the average annual attack rates and secondary attack rates compared to the population with a one-child policy.

One limitation of our findings is that it is impossible to know what would have happened in the absence of the one-child policy. Because our goal was to highlight the role of household size and other related demographic changes, we simply assumed an extrapolation from 1970s trends. In reality, demographic changes may have occurred for other reasons in the absence of a one-child policy. Moreover, this model did not distinguish contacts other than household and school (for example workplace [71], [72], [73], or community [74]). Containment measures, such as different vaccine strategies [75], [76] and travel restrictions [77], [78], were not considered in this model, allowing for a focus on the relationship between child policies and influenza transmission. We did not distinguish antigenic diversity [79]; because aging populations have more cross-immunity for similar strains [8]. This limitation may underestimate an aging effect on influenza transmission. All parameters used in this model were defined from existing published literature. We did not assess the differences between pandemic years versus inter-pandemic years because of the assumption that there are no changes in influenza natural history parameters during the course of over 30 years. We did not use this model to answer an important question that whether or not the demographic changes affect pathogen emergence in China because of lacking sufficient data, and this question is beyond the scope of this paper.

This study found that the average annual attack rate is slightly lower in a population with a one-child policy, which may result from a decreased household size (from 4.2 in 1979 to 3.5 in 2004 in the model) and the decreased proportion of children (who are more vulnerable to infection than adults) in the population because of the dramatically reduced fertility rates from 2.9 in 1979 to 1.3 in 2004. However there is no discernible difference in the *SAR*. A possible reason for the absence of a discernible difference is that the decrease of average household size (from 4.2 to 3.5) might not be large and fast enough to obviously reflect the change in the secondary attack rate. We compared the results of this study with other recent studies [61], [80], [81], [82], [83] about the relation between household size and *SAR*, household size and the overall attack rate. The lower annual attack rate with smaller household size is consistent with the results from Fraser et al. [61] and Kwok et al. [83], but Carcione et al. [81] found that individual risk was not associated with the household size. The absence of a discernible difference in the *SAR* observed in this study is similar to the findings in [80] in which the *SAR* remained stable as household size increased, while the *SAR* increased with larger household size in other studies [61], [82], [83]. The above comparisons included some studies in which the *SAR* was measured empirically, though the relation between the simulated *SAR* and household size may be controlled by the model structure. In this model, the *SAR* was estimated by the proportion of household contacts of an index case who subsequently became infected [62], [64], so that the simulated *SAR* stands in relation to the simulated epidemic, which is in the same way the real-world empirical *SAR* and its relation to the true unobserved epidemic.

## Supporting Information

Figure S1 **Model structure.**
(TIFF)

Figure S2
**Progression of the model.** Given a time *t*, each individual in the model is in one state of 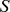 (susceptible), 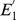 (mild exposure), 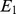 (not mild exposure), 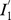 (mild asymptomatic infectiousness), 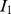 (not mild asymptomatic infectiousness), 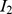 (symptomatic infectiousness) and 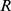 (recovered with immunity), and the population's inflow and outflow are represented by each individual's age-specific death rate *d* and age-specific fertility rate *b*.(TIFF)

Figure S3 **Demographic calibration.** (A) Population projection using census data and a Leslie matrix. Case 1: population trajectory based on static data of survival probability (*sv*) and maternity rate of female (*mf*) in 1975. Case 2: population trajectory based on dynamic data of *sv* and *mf* in 1975, 1982, 1990, 2000 and 2009. Case 3: population trajectory based on dynamic data of *sv* and *mf* in 1975, 1982, 1990 and 2000. Case 4: population trajectory based on dynamic data of *sv* and *mf* in 1975, 1982 and 1990. Case 5: population trajectory based on dynamic data of *sv* and *mf* in 1975 and 1982. Case 6: population trajectory based on dynamic data of *sv* and *mf* in 1982, 1990 and 2000. Case 7: population trajectory based on dynamic data of *sv* and *mf* in 1982, 1990, 2000 and 2009. * The trajectory from Song J, Yu J (1988) Population system control: Springer. Note: this population projection did not include influenza transmission. After the population calibration, the simulations for influenza transmission only included 10,000 initial population. (B) Age structure. The solid lines in blue, red, green, orange, and purple are the simulated proportions in age groups 0 to 4, 5 to 9, 10 to 25, 26 to 49, and 50+, respectively. The dotted lines in the same colors are the observed proportions of the five age categories from census data. (C) Average household size. The blue, green and red lines are simulated average household sizes of each year under three scenarios: one-child policy, strict one-child policy, and absence of one-child policy. With a one-child policy (the blue line), the simulated average household size decreased from 4.2 in 1979 to about 3.5 in 2009, which is similar to the change in average household sizes reported in the census data: 4.43 (in 1964), 4.42 (1982), 3.96 (1990) and 3.44 (2009).
(TIFF)

Text S1 **Supplement.**
(DOC)

Table S1 **The estimated** ***AR*** **and** ***SAR*** **.**
(DOC)

Table S2 **Some articles of influenza in China.**
(DOC)
