# Supplementary figures and images for: Effect of the One-Child Policy on Influenza Transmission in China: A Stochastic Transmission Model
Source: PLoS One. 2014 Feb 6;9(2):e84961. doi: 10.1371/journal.pone.0084961 (PMC3916292; doi:10.1371/journal.pone.0084961)

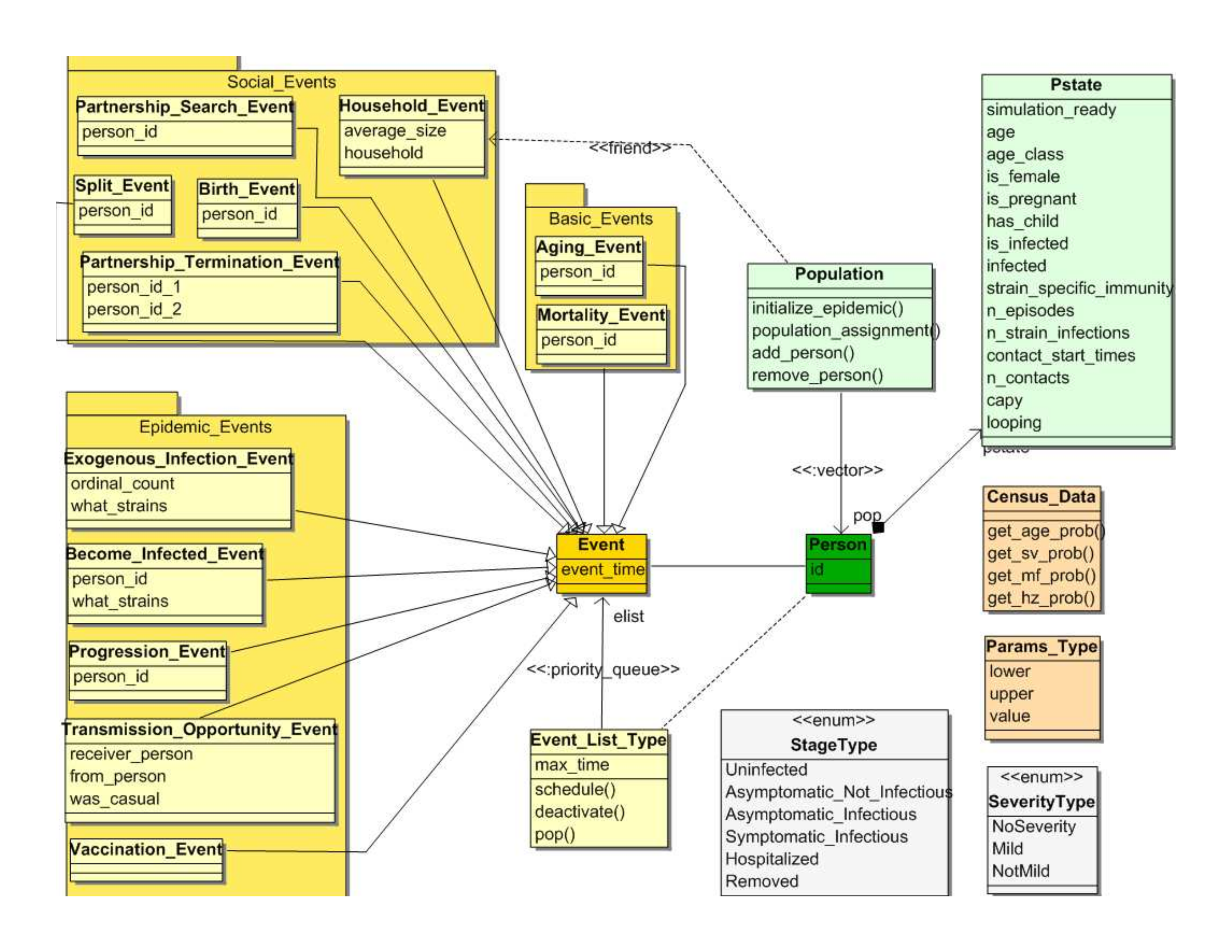

Supplement: Figure S1 — Model structure. (TIFF) [file pone.0084961.s001.tiff]

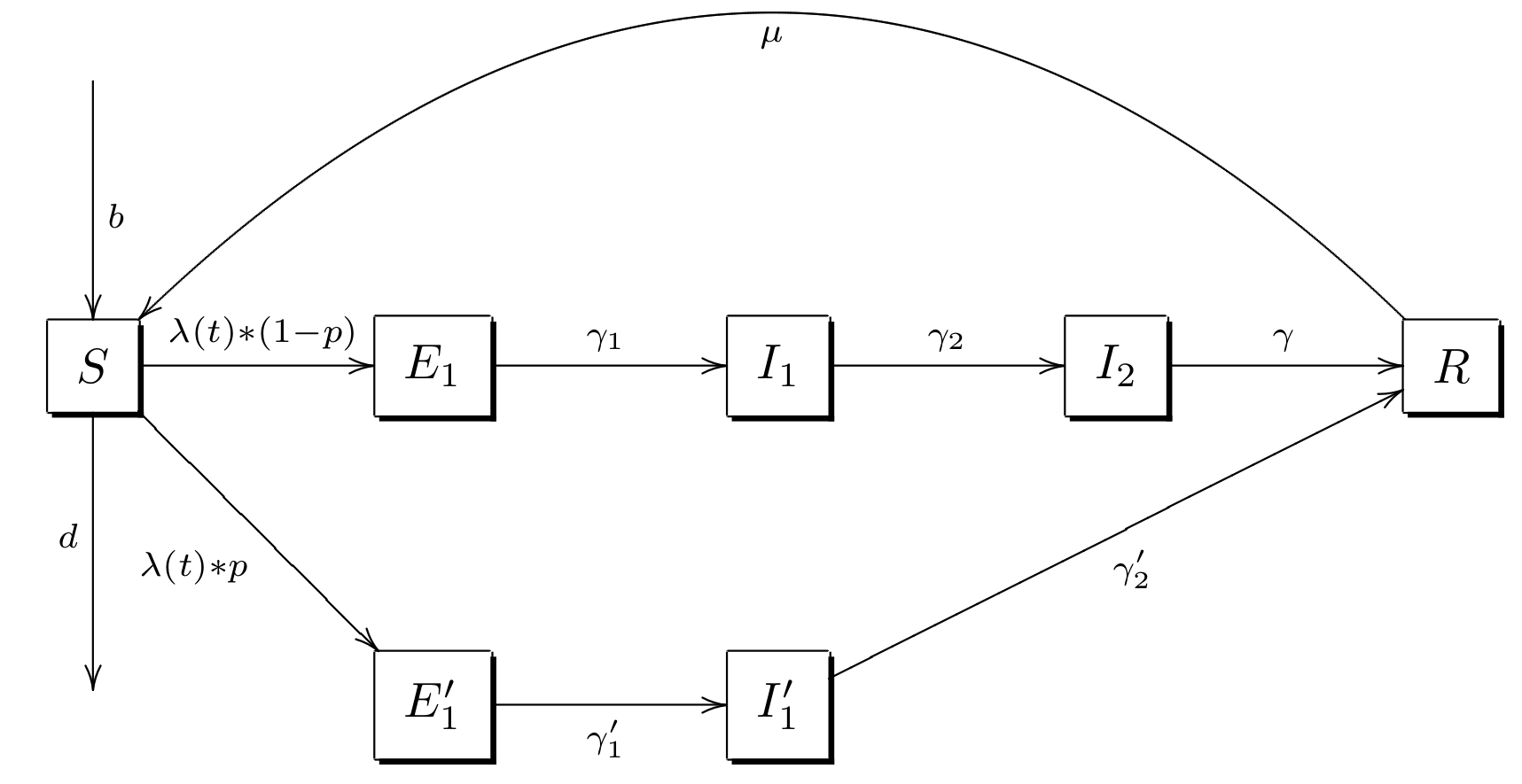

Supplement: Figure S2 — Progression of the model. Given a time t, each individual in the model is in one state of (susceptible), (mild exposure), (not mild exposure), (mild asymptomatic infectiousness), (not mild asymptomatic infectiousness), (symptomatic infectiousness) and (recovered with immunity), and the population's inflow and outflow are represented by each individual's age-specific death rate d and age-specific fertility rate b. (TIFF) [file pone.0084961.s002.tiff]

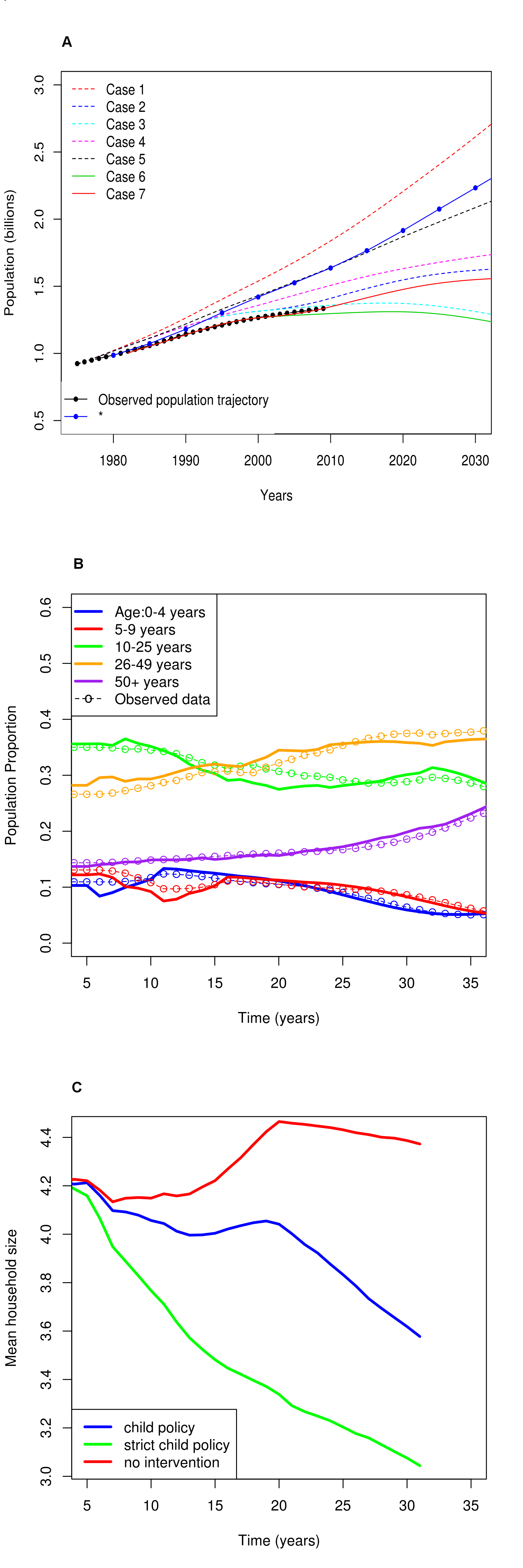

Supplement: Figure S3 — Demographic calibration. (A) Population projection using census data and a Leslie matrix. Case 1: population trajectory based on static data of survival probability (sv) and maternity rate of female (mf) in 1975. Case 2: population trajectory based on dynamic data of sv and mf in 1975, 1982, 1990, 2000 and 2009. Case 3: population trajectory based on dynamic data of sv and mf in 1975, 1982, 1990 and 2000. Case 4: population trajectory based on dynamic data of sv and mf in 1975, 1982 and 1990. Case 5: population trajectory based on dynamic data of sv and mf in 1975 and 1982. Case 6: population trajectory based on dynamic data of sv and mf in 1982, 1990 and 2000. Case 7: population trajectory based on dynamic data of sv and mf in 1982, 1990, 2000 and 2009. * The trajectory from Song J, Yu J (1988) Population system control: Springer. Note: this population projection did not include influenza transmission. After the population calibration, the simulations for influenza transmission only included 10,000 initial population. (B) Age structure. The solid lines in blue, red, green, orange, and purple are the simulated proportions in age groups 0 to 4, 5 to 9, 10 to 25, 26 to 49, and 50+, respectively. The dotted lines in the same colors are the observed proportions of the five age categories from census data. (C) Average household size. The blue, green and red lines are simulated average household sizes of each year under three scenarios: one-child policy, strict one-child policy, and absence of one-child policy. With a one-child policy (the blue line), the simulated average household size decreased from 4.2 in 1979 to about 3.5 in 2009, which is similar to the change in average household sizes reported in the census data: 4.43 (in 1964), 4.42 (1982), 3.96 (1990) and 3.44 (2009). (TIFF) [file pone.0084961.s003.tiff]
