# Supplementary material for: Effect of the One-Child Policy on Influenza Transmission in China: A Stochastic Transmission Model
Source: PLoS One. 2014 Feb 6;9(2):e84961. doi: 10.1371/journal.pone.0084961 (PMC3916292; doi:10.1371/journal.pone.0084961)
Supplement: Text S1 — Supplement. (DOC) [file pone.0084961.s004.doc]

**Text S1: Supplement for “Effect of the one-child policy on influenza transmission in
China: a stochastic transmission model”**

Fengchen Liu^1,2^, Wayne T. A. Enanoria^1,2,3^, Kathryn J. Ray ^1^, Megan P. Coffee^1,2^, Aubree
Gordon^3^, Tomás J. Aragón^2,3^, Guowei Yu^4^, Benjamin J. Cowling^5^, Travis C. Porco^1,2,6^*

^1^F.I. Proctor Foundation, University of California, San Francisco, California, USA

^2^Center for Infectious Diseases and Emergency Readiness, School of Public Health, University of California, Berkeley, California, USA

^3^Division of Epidemiology, School of Public Health, University of California, Berkeley, California, USA

^4^West of China Institute of Environmental Health*,* Northwest University for Nationalities, Gansu, China

^5^School of Public Health, The University of Hong Kong, Hong Kong

^6^Department of Epidemiology & Biostatistics, University of California, San Francisco, California, USA

* To whom correspondence should be directed: T Porco, FI Proctor Foundation, University of California, 95 Kirkham Avenue Box 0412, San Francisco, California 94143; [travis.porco@ucsf.edu](mailto:travis.porco@ucsf.edu); Ph. 415 476-4101; FAX 415 476 0527.

9 January, 2014

# 1 Introduction

In this Appendix, we provide full details about the model structure and parameters we used to simulate influenza transmission in the population with demographic control policies.

# 2 Overview

## 2.1 Model Structure

We used an individual-based modeling and simulation approach to model influenza in China , following the approach of previous transmission studies . Our model has two basic elements: 1) a set of individuals, each individual possessing attributes and behaviors, and 2) a set of individual relationships and methods of interaction, which define how and with whom individuals interact .

In our model, these two elements were illustrated in Figure S1. As an individual, every “Person” has detailed attributes (a class of attributes of individual) describing the state of the individual; state changes are referred to as events. Possible (“active”) events are associated with event times; the simulation proceeds by choosing the active event with the earliest active time (in an event-driven simulation). The state variables describing each person can be static (i.e., not changeable during the simulation, such as identification and name of a person) or dynamic (i.e., changeable as the simulation progresses, such as contact links in household, school and community, or different states during the course of an infection). The population is simply a collection of persons.

## 2.3 Formal Specification

We describe the specification of the continuous-time, discrete-event system using the terminology and theory of . We specify five components: (1) the state space of the model, (2) the set of possible events, (3) the set of active events of each possible state, (4) the transition function which specifies how the state of the system changes when an event occurs, and finally (5) the parameter values.

Our model can be formalized as a generalized semi-Markov scheme (GSMS) of a discrete-event system . In a population with *N* individuals, we assume that there are *k* state types for each individual, a set of state types is$Y=\{Y_{1},Y_{2},\cdots,Y_{k}\}$. For example, the state types for individual *j* is denoted $Y_{j}=\{Y_{j,1},Y_{j,2},\cdots,Y_{j,k}\}$. Then we have the state space (Cartesian product) of individual *j*, $X_{j}=Y_{j,1}\times Y_{j,2}\times\cdots\times Y_{j,k}=\{X_{j}^{(1)},X_{j}^{(2)},\cdots,X_{j}^{(m)}\}$, where $m=\prod_{i=1}^{k} \left|Y_{j,i}\right|$, and each $x\in X_{j}$ is a specific state of individual *j*. Therefore, the state of the population as a whole system can be represented by the states of all the individuals. We assume $S$ as the state space of the population, then$S=X_{1}\times X_{2}\times\cdots\times X_{j}\times\cdots\times X_{N}=\{s_{1},s_{2},\cdots,s_{n}\}$, where$n=\prod_{j=1}^{N} \left|X_{j}\right|$, and each $s\in S$ is a unique state of the population. For each$s\in S$, there is a set of active events ($E(s)$), each active event $e\in E(s)$ can change the state of one or more individuals in the population with state$s$. With the action of event$e$, state$s$could move to another different state$s'$with probability$p$,$p(s';s,e)$. The universal event$A=\bigcup_{s\in S} E(s)$ constitutes an event set for$S$. According to the definition of GSMS in , the population state space$S$and event set$A$(as we described above) construct a GSMS. In this population system, when we specify an initial state$s^{0}$, we assume that all other states in$S$are reachable from$s^{0}$ as follows: (1) for every$s\in S$, there exist states$s^{1},\cdots,s^{r}$and events$e^{0},e^{1},\cdots,e^{r}$ with$e^{i}\in E(s^{i})$,$i=0,1,\cdots,r$, and (2) $p(s^{1};s^{0},e^{0})p(s^{2};s^{1},e^{1})\cdots p(s;s^{r},e^{r})>0$. That means any state in this population can be reached by any other state by changing some individuals’ states.

Simulation proceeds as follows. Based on the overall population state, certain events are possible (“active”). For instance, if an individual is latently infected, it is possible for that person to become contagious to others in the future. Or, if a person is infectious, it is possible for that person to actually infect others, as well as to recover with partial immunity. Each active event has an event time associated with it; the simulation proceeds by choosing the active event with the earliest time. The state of the system is then transformed according to the chosen events , and this simulation time, *t,* is updated to the time *t*+1. This process yields a sequence of event occurrence times $t^{0},\cdots,t^{q},\cdots$(for $0\leq i\leq q$), and a corresponding sequence of the state values at these times$s^{0},\cdots,s^{q}\cdots$, for each event time index$q=0,1,\cdots$. Thus, $s^{0}$ is the initial condition of the system at time$t^{0}$; the first event occurs at time $t^{1}$ and causes the state of the system to change from$s^{0}$ to $s^{1}$, and so forth. When such a state change takes place, new events may become active (and their event times must be determined and rescheduled); other events may no longer be active (and they must be removed from the event list). We first specify the state $S$ of the system, and then each of the possible events.

# 3 State Specifications

## 3.1 State of Individual

We first specify the possible states of individuals in the model. Individuals are indexed by *j*, *j* = 1,2, …, *N*(*t*), where *N* is the number of individuals at time *t*. For each individual *j*, if the individual is alive, the dead indicator *D_j_* = 0, otherwise *D_j_* = 1. Uninfected individuals have an influenza stage *G_j_* of *S*; the stages are indicated in Figure S2. It is convenient to define *I_j_* as an indicator of whether person *j* is infectious, i.e. *I_j_* is 1 (if *G_j_* is *I*_1_, *I*_2_, or *I*’_1_), and 0 otherwise; also, let *Y_j_* indicate whether individual *j* is infected but not infectious, i.e. *Y_j_* is 1 if *G_j_* is *E*_1_ or *E’*_1_, and 0 otherwise. Finally, we define *Q_j_* as an indicator of whether person *j* is in the recovery phase, i.e. whether *G_j_* is *R* or not. For individual *j*, some important individual states at time *t* include: (1) severity at time *t*, *S_j_*(*t*); (2) influenza state at time *t*, *G_j_*(*t*); (3) immunity status at time *t*, *M_j_*(*t*); (4) household contacts at time *t*, *H_j_*(*t*); (5) school contacts at time *t*, *Sch_j_*(t); (6) casual contacts at time *t*, *C_j_*(*t*); (7) number of children at time *t*, *B_j_*(*t*).

**Severity.** Once an individual is infected, the influenza severity indicator *S_j_* is defined to be MILD or NOT MILD, which follows a binomial distribution with the probability of being a mild case, *p_mild_* (please see Table 2 in the main text for details of this parameter).

**Influenza Stage.** For individuals with mild influenza (*S_j_* = MILD), we assume the following stages. We assume that for individual *j* who has never been infected, the stage *G_j_* = *S*; if this individual is infected, then the individual will go through *G_j_* = *E’_1_* (infected prior to all symptoms and infectiousness), *G_j_* = *I’_1_* (infectious but asymptomatic), then *G_j_* = *R* (recovered). For individual with a non-mild case, (*S_j_* = NOT MILD), the individual *j* has *G_j_* = *S,* *E*_1_, *I*_1_, *I*_2_, or *R*, where *I*_2_ is stage of symptomatic infectiousness, *E*_1_ and *I*_1_ have the similar definitions as *E’*_1_ and *I’*_1_ for mild cases, respectively. The durations that *G_j_* = *E’_1_*, *E_1_*, *I’_1_,* *I_1_* and *I_2_* are exponentially distributed random variables with rates of *γ’*_1_, *γ*_1_, *γ’*_2_, *γ*_2_ and *γ* (respectively); these are reciprocals of the latent period for a mild case, the latent period for a non-mild case, the asymptomatic infectious period for a mild case, the asymptomatic infectious period for a non-mild case, and the symptomatic infectious period for a non-mild case, respectively (please see Table 2 in the main text for details of these parameters).

**Immunity Status.** Influenza viruses are divided into subtypes based on differences in hemagglutinin (HA) and neuraminidase (NA) . There are 16 antigenically different HA subtypes and 9 antigenically distinct NA subtypes, which in combination define all known subtypes of influenza A viruses . While the influenza surface HA glycoprotein is the antigenic target, the virus is capable of evading immune recognition through continual antigenic drift of its surface glycoproteins, HA and NA . The H3N2 sequences fall into groups or clusters with unique antigenic properties . There is almost complete immunity between strains within a same cluster, and cross-immunity is as low as 60% to 85% between different clusters . We assume that an individual *j* has a dynamic immunity level$M_{j}(t)$ during the simulation:

$M_{j}(t)=\left\{\begin{matrix} m_{base},t=0 \\ m_{full},G_{j}(t)=R \\ M_{j}(t-\Delta t)(1-\mu),0\leq\mu\leq1 \end{matrix}\right.$ (1)
where the *m_base_* is the mass immunity at initial time, *m_full_* denotes the highest immunity level of the individual at the time of his/her recovery, and *μ* is immunity loss rate. Equation (1) means that the immunity of an individual reaches the highest level immediately after recovery, and then gradually decreases until next infection.

**Household Contacts.** Each individual *j* in the model is associated with a list of household contacts (*H_j_*). The household links *H_j_* are initialized using the population data of China in 1975, and are updated dynamically (1) when the individual leaves his or her household between his/her age 14 and 18 years as a single household, (2) when the individual (if single and age > 18) has found another single (age > 18) to live with, thereby initiating a new two member household, (3) at the time the individual dies, or (4) at the time the individual or one of the other family members gives birth.

**School Contacts.** Each individual *j* whose age is between the primary-school-age 6 and 12 years or the middle-school-age 13 and 18 years has a vector to hold the links to each of his/her other schoolmates (*Sch_j_*). The school links *Sch_j_* are initialized using the primary and middle schools’ statistical data of Gansu province in China in 1975, and are updated annually by reassigning all individuals with school ages in primary or middle schools according to the average school size of each year from 1976 to 2009. The school links are also updated at the time of death of the individual. Like the household contact network, the school contact network is a directed graph, so a school link update between individual *j* and one of its schoolmates *i* means updating two school links from *j* to *i* and from *i* to *j*.

**Casual Contacts.** Each individual *j* may have several random contacts per day with a daily contact rate *contact_casul_* =16. Once individual *j* becomes infectious, all of his or her casual contacts during the infectious period are predicted using an exponential distribution, and saved in a vector (*C_j_*) which will be emptied at the time of recovery or death of the individual *j*, and updated once a casual contact of j is dead.

**Number of Children.** For a female individual *j*, *B_j_* **=** 0, 1, …, *k* is the number of living children she already has. Depending on the value of the child policy indicator *Z*, *B_j_* can be used to decide whether or not the individual is allowed to give birth. For example, if a strict one-child policy is active, then *B_j_* < 1 is the precondition for giving birth; if the two-child policy is active, she will be allowed to give birth as long as *B_j_* at current time *t* is less than 2.

## 3.2 Global Variables

Additionally, *Z* is defined as the child policy indicator. In the absence of any fertility control policy (*Z* = 0), the model uses the age-specific fertility rate and death rate from census data in 1975 as static population’s fertility and death rates during the simulation. If *Z* = 1, we simulate assuming the one-child policy; the model will use dynamic age-specific fertility rates and death rates from year 1975 to 2009 to simulate the population growth under scenarios of the one-child policy. When *Z* is 2, a very strict (counterfactual) one-child policy allows one female to have only one child in her life. When *Z* is 3, the two-child policy (from 2015 to 2024) allows one female to have two children since 2015; this is implemented by increasing the fertility rate for females who have not had a child yet.

Exogenous infection, *K*, is defined as the number of individuals who are exogenously infected to initiate the epidemic of influenza in the population. The exogenous infection period, *θ*, is the time point of each year at which the *K* infectious exogenous are periodically introduced into the population.

## 3.3 Full State Space

The full state space ***X****_j_* for an individual in the system is specified by the collection
***X_j_*** = { *G_j_*, *S_j_*, *Y_j_*, *Q_j_*, *M_j_*, *D_j_*, *H_j_*, *C_j_*, *B_j_*, *P_j_ μ, Z, K, θ, p_mild_, γ’_1_, γ_1_, γ’_2_, γ_2_*, *γ* },
where *G_j_* denotes the collection of influenza stage values for each individual *j*, etc. The full state space ***S*** for the population system is specified by the collection of each individual’s state space: $S=X_{1}\times X_{2}\times\cdots\times X_{j}\times\cdots\times X_{N}=\{s_{1},s_{2},\cdots,s_{n}\}$, where$n=\prod_{j=1}^{N} \left|X_{j}\right|$, and each $s\in S$ is a unique state of the population.

## 3.4 Initial Conditions

The model is initialized with *N*(0) individuals whose ages are generated from the age distribution of China’s population in 1975. We assume that all individuals are alive (*D_j_* = 0 for all *j*), totally susceptible (*S_j_* = 0 and *G_j_* = 0 for all *j*), and have no immunity (*M_j_* = 0) at the beginning of simulation, and a 10% immunity loss rate per year (*μ* = 0.1) after infection. Household links *H_j_* for each individual are generated from the household size distribution of demographic data of China in 1975. School links, *Sch_j_*, for each individual of school age are assigned to schools using the average school size of Gansu province in China in 1975. Links outside of the household for each individual, *C_j_*, are updated each day based on the daily casual contact rate (*contact_casual_* = 16). Five exogenous infections are introduced into the population on November 15^th^ in each year (*K*= 5, *θ* = 318). The scenario with a population control policy (e.g., one-child policy) is one of four levels (*Z* = 0,1, 2, 3).

The initial population of the year 1975 was generated using four steps: 1) initialization of household size and age structures, 2) mother-child relationship initialization, 3) household links initialization, and 4) school links initialization.

### Household size and age structure initialization

Each individual’s age and gender were initialized according to the age distributions and sex ratio in 1975. To fit the model to the observed household size distribution in 1975, i.e., a household with members of 1, 2, 3, 4, 5, 6, 7, or 8+ had a probability of 0.08, 0.10, 0.16, 0.19, 0.18, 0.13, 0.07, or 0.09 (respectively); we first used this distribution to assign every individual to a household, and then we used the Metropolis-Hastings (MH) algorithm to initialize the population by arbitrarily switching two individuals from different households (this step was repeated 100,000 times to increase the total score of households). The households’ score used in the MH algorithm was calculated based on following cases:

(1) For a 1-member household, a single adult household has a higher score than a single child household.
(2) For a 2-member household, the score decreases in the following order: one male adult and one female adult, one adult and one child, two male or two female adults, and two children.
(3) For a 3-member household, the following are in order of decreasing score: one male adult and one female adult and one child, one adult and two children, two male/female adults and one child, three adults, and three children.
(4) For a 4-member household, the following are in order of decreasing score: one male adult, one female adult, and two children; two male adults and two female adults; two male adults and two children, or two female adults and two children; three adults and one child; one adult and three children; four adults, and finally four children.
(5) For a 5-member household, the following are in order of decreasing score decreases with a household structure of one male adult and one female adult and three children, one male adult and one female adult and one male/female adult and two children, three male/female adults and two children, four male/female adults and one child, two male adults and three female adults, three male adults and two female adults, one adult and four children, five adults, and five children.
(6) For a 6-member, 7-member and 8-member households, the scores decreases with the household structure from the most common household structure to the less common household structure (as described above).
(7) For a household with 2 or more members, it has a higher score if the oldest child is at least 16 years younger than the oldest adult.

Mother-child relationship initialization

Based on the initialized population stated above, the mother-child relationship was initialized as follows: 1) for each household, we randomly assigned each child a female household member, whose age was between 16 to 49 years and at least 16 years older than the child’s age, as the child’s mother; 2) if step 1) did not assign every child a mother, we randomly chose a woman (at least 16 years older than the child, and between age 16 and 49 years) in the population to be his/her mother.

To test whether or not these initialized mother-child relationships were consistent with the census data, we computed the simulated fraction of mothers with at least 1 alive child in women between the ages of 16 and 49 years, , and the expectation of this fraction based on the observed age-specific fertility rates and survival probability of children at age 0 in 1975, , where $n_{sim}$ is the total number of runs, $m_{a}^{k}$ is the *k*th simulated total number of mothers at age $a$ and with at least 1 alive child, $f_{a}^{k}$ is the *k*th simulated total number of women at age $a$, $\phi_{i}$ is the observed fertility rate at age $i$ in 1975, $s_{0}$ is the observed survival probability for children at age 0 in 1975, $\delta_{a\geq31}$ is an indicator function whose value is 1 if age $a$ >= 31 and 0 otherwise, and$\delta_{a<31}$ is an indicator function that has value of 1 if $a$ < 31, otherwise its value is 0.

We computed $\bar{\rho}*$ and estimated$\rho*$ based on a simulation with 400 replications, and found that the simulated fraction of mothers with at least 1 alive child in women between age 16 and 49 years, $\bar{\rho}*$= 0.628, was close to the expected fraction $\rho*$= 0.599.

Household links initialization

For each initialized household, every individual in the household was linked to all other household members as his/her initial household links.

School initialization

The total number of primary schools was decided from the total number of children between age 6 to 12 years and the average primary school’s size in 1975. Similarly, the total number of middle schools was equal to the total number of children between ages 13 to 18 years divided by the average size of middle school in 1975. Every primary school-age student and every middle school-age student were randomly assigned to an available primary school and an available middle school. For each school, every student was linked to all other students in the same school as that student’s initial school links.

The epidemic was initiated by randomly selecting *K* individuals and scheduling “become infected events” (see below) for them at time *t^0^* = 0, the beginning of the simulation.

## 3.5 Calibration of Transmission Parameters

The calibration of transmission parameters was done by using Approximate Bayesian Computation to fit the average annual attack rate (*AR*) within the range (0.1, 0.2), and secondary attack rate (*SAR*) inside the range (0.09, 0.32). For details of the *AR* and *SAR* we cited, please see Table S1.

In order to obtain empirical data of influenza in China, we searched PubMed on January 30, 2013 using the MeSH Terms: **Influenza, Human**AND**China**AND**epidemiology**, and found 929 articles. We selected 174 articles from 929 articles to review. We categorized 76 of the 174 articles into influenza-like illness (ILI), SAR, AR, etc. as shown in the Table S2 (additional 82 articles were updated by searching the same keywords in PubMed on July 22, 2013). The available data from most of these articles were sentinel data on ILI; the SAR and AR reported by some articles were already included in the ranges of SAR and AR we used for parameter calibration.

# 4 Events

The set of important events are shown in Figure S1. The event times and transitions associated with each of these events will be discussed in turn. Any state variables (components of **X**) whose values are not otherwise specified for any particular event are assumed to remain unchanged from **X***^k^*. We assume three types of events: basic events, social events, and epidemic events. Events of the basic type change the individual’s attributes such as age and death. Social events update the contact networks when an individual leaves a household after growing up to be single, when an individual has found a partner to live with as a 2-member household. Epidemic events include behaviors that spread influenza via the contact networks, and progression to update the health status of individuals.

## 4.1 Basic Events

### 4.1.1 Aging Event

For each individual *j*, if the age of that individual, denoted *Age_j_*, is less than the assumed maximum possible age (120 years) and the individual survives to the next year based on the observed mortality rate of *Age_j_* in the current year, *Age_j_* is increased by 1. Otherwise, the Mortality Event (see 4.1.2) will be called to remove the individual from the population, and update all information related to the removed individual. The Birth Event (which will be discussed below) will be scheduled for the female individual in the next 9 to 12 months depending on the policy indicator *Z* and number of children of woman *j*, *B_j_*. Specifically, the policy indicator may correspond to (0) no demographic policy, (1) the one-child policy as realized, (2) the strict one-child policy (a hypothetical one-child policy in which no exceptions occur—no female ever has more than one child), and (3) a hypothetical future strict two-child policy beginning in 2015, in which two children may be borne by each female, but never more than two. The Split Event (see 4.2.3) will be called for an individual, who is living with other family members in a household and whose age is between 14 and 18 years, when that individual is reassigned as an individual of a 1-member household. If the single individual whose age is over 18 years, the Partnership Search Event will be scheduled for the individual to start looking for another single to live with as a 2-member household. Finally, a new Aging Event will be scheduled for the survived individual on his/her next birthday. And this process will repeat for each individual until a mortality event or the end time of the simulation.

We model demographic policies as follows. First, we assume a counterfactual age-specific fertility rate *F_ya_* for year y and age class a; this rate will be used to provide an initial estimate for the number of births for each woman each year. The counterfactual rate determines what the number of births per year would be in the absence of policy-specific restrictions on the number of children a woman may bear. Then, during the simulation, we then modify this based on the number of children a woman has had. Thus, the actual simulated fertility rate is determined by the assumed counterfactual fertility rate as well as the population history and demographic policy. The counterfactual fertility rate may be considerably higher than the simulated fertility rate once each woman’s fertility history has been included. The specific policy models are as follows:

- No demographic policy:
  When we assume that no demographic policy is in place, we assume that the probability of giving birth in a given year for a female of a given age is determined by the counterfactual age-specific fertility rate, *F_ya_*. Moreover, we always choose the rate implied by the 1975 census in order to extrapolate the conditions prior to the one child policy. That is, we use the 1975 life table as the counterfactual fertility rate, and we do not modify the number of children each woman has per year based on her fertility history. While such a demographic trajectory would have been unsustainable, it serves as a basis for comparison of influenza scenarios.
- One-child policy as realized:
  When we assume the one-child policy as realized, we note that the policy permits certain exceptions (e.g., in rural areas and for ethnic minority populations), and that compliance may not have been perfect. We assumed that women who already had one child could have a second child, with probability *η*, which was unknown and needed to be calibrated from the range 0% to 100%. For a given female *j* in the simulation, we determined whether or not she would have a birth during year *y* as follows. We first determined, using an assumed counterfactual age-specific fertility rate, *F_ya_* (for age *a*, year *y*), the probability of a possible birth during the year (the fertility rate that would occur in the absence of specific restrictions). For each female and each year, a single Bernoulli trial was conducted with this probability. If this trial was a success, a birth was scheduled for the year if the woman had no previous children. If the woman had at least 1 living child, then we determined if an exception to the one-child policy occurred. This was determined by drawing an additional Bernoulli trial using the exception probability for the current year. The simulation does not consider rural versus urban, or ethnic status, explicitly.
- Strict one-child policy:
  When we assume the strict one-child policy, we assume that the probability of giving birth in a given year (1975 to 2009) for a female of a given age is determined by the counterfactual age-specific fertility rate, *F_ya_*, for females who have no living children; if a female already has living children, then her fertility rate is assumed to be 0. This is identical to the previous policy, assuming *η*=0.
- Two-child policy:
  We assume this hypothetical future strict two-child policy from 2015 to 2024, in which two children may be borne by each female, but never more than two. That is, in the simulation, the one-child policy as realized is applied into the population from 1975 to 2014; from 2015 to 2014, a woman is allowed to give birth with her age-specific fertility rate as long as the number of previous children for this woman is less than 2. Possible children are simulated using the counterfactual age-specific fertility rate *F_ya_*; only if the mother has fewer than two living children are the births realized.

The calibration for the counterfactual fertility rate, *F_ya_*, is based on assuming that the simulated age specific fertility rate agrees with estimate from the census data. That is the counterfactual rate, when combined with the population history and policy, should yield a simulated fertility rate in approximate agreement with the observed data.

To calibrate the counterfactual rates *F_ya_*, we used the following procedure. First, age-specific fertility rates *f_ya_* consistent with the observed demographic trajectory were computed from successive decennial census figures using standard methods. Simulation based on the values of *f_ya_* alone (without considering the individual history of each female) yields demographic trajectories in approximate agreement with the census observations by design. Second, the values for the time-dependent exception probability *η_y_* were randomly chosen from 0 to 1. Third, we multiplied the given age-specific fertility rates, *f_ya_*, by 1+ *ξ_y_* (1− *η_y_*) to yield an initial trial value for *F_ya_*. Fourth, we simulated the model over the entire time course 100 times to determine the simulated age-specific fertility in the presence of the policy. Finally, we iterated this process, correcting the trial fertility possibility rates (by adjusting the value of *ξ_y_* from 0 to1) until the simulated age-specific fertility matched calculations based on the successive censuses.

To calibrate the value of *F_ya_* , we must use the value of *F_ya_* in the model, together with the individual history of each female in the simulation. We choose a value of *F_ya_* such that the simulated fertility rate is approximately given by *f_ya_* (and is therefore consistent with the census data). Our algorithm is simply based on multiplying the value of *f_ya_* by a value *ξ_y_ and a value η_y_.* Thus, we must compute two numbers (*ξ_y_* and *η_y_*, and then show that the simulated fertility rates approximately equal *f_ya_*). We do not assume that there exists a unique best value for the values *ξ_y_* and *η_y_*. Simply using the census-based fertility rates *f_ya_* in place of the counterfactual rates *F_ya_* may yield simulated fertility rates which differ from the census-based rates *f_ya_* because of the simulated effect of the policy in preventing births which might otherwise have occurred.

We assess the discrepancy between the simulated age-specific fertility rates from the model and the census fertility rates *f_ya_* (based on census data from 1975 to 2009) using a measure based on both the sum of the squared differences between the simulated values and the census values, but also an additional term measuring the difference between the ranks. Thus, some importance is given to ensuring that the age group with the largest estimated fertility rate implied by the census data corresponds to the age group with the highest simulated fertility. Specifically, the discrepancy is given by , where *a* indicates a five-year age group (15-19, 20-24, etc.), *R*_ya_ is the rank of the observed fertility rate of the *a*th age group among all of the age groups based on observed age-specific fertility rates at year *y*, is the rank of the simulated fertility rate of the *a*th age group among all age groups based on simulated age-specific fertility rates at year *y*, and the arbitrary parameter *λ* serves to weight the importance of the squared error component and the rank component $|\hat{R}_{ya}-R_{ya}|^{2}$ of the overall discrepancy measure. Thus, given any input parameters (in particular, any choice of the parameters *ξ_y_* and *η_y_*, that (together with *f_ya_*) determine the counterfactual fertility rate *F_ya_* and any choice for the demographic policy), the simulation can be repeatedly executed, to obtain an estimate for, from which the discrepancy may be computed. Note that by averaging the results of many simulations, the Monte Carlo error in may be rendered small.

To conduct the calibration, we used a simple ad hoc grid search as follows. We selected values of *ξ_y_* and *η_y_*, ranging from 0 to 1 in steps of 0.04, yielding 676 grid points defining 625 small rectangles with length 0.04 on each side. For each grid point, we conducted 100 replications to estimate the discrepancy for each of the 676 scenarios. We chose the grid point with the smallest discrepancy (where *ξ_y_* from 0 to 0.04 and *η_y_* from 0.74 to 0.78), assuming somewhat arbitrarily a value for λ so that the rank terms in the discrepancy are approximately one tenth the size of the squared error term.

### 4.1.2 Mortality Event

A mortality event removes an individual *j* from the population, deletes its household links *H_j_* , school links *Sch_j_* and casual links *C_j_*, and notifies all of its household members in *H_j_* and schoolmates in *Sch_j_* to delete their links to the removed individual *j* from their contacts lists, and it finally deactivates and removes all events associated with the removed individual.

## 4.2 Social Events

### 4.2.1 Household Event

At the beginning of the simulation, the household event initializes the households by assigning all individuals into different households according to the household size and the age distributions from the 1975 China census data. During the simulation, it updates household information (household size and members of each household) based on individuals’ household links, and reports the household information every year. It always reschedules itself for the next year until the end time of the simulation.

### 4.2.2 Birth Event

For mother *j*, a new baby *i* is added into the population at the time of birth event with initialized attributes (age = 0, gender = F or M, immunity = 0), *B_j_* is increased by 1, the new baby *i* will link to mother *j* and each of her household members in *H_i_* as the new baby’s household links *H_i_*. Meanwhile, *j* and all other family members in *H_i_* need to add one more household link to the new baby *i.* The birth event finally schedules the aging event for the new baby on the same day in the next year.

### 4.2.3 Split Event

An individual whose age is between 14 and 18 years has a chance to leave the individual’s current household to be a single household. Once the split event happens for individual *j*, it has to update the links of household *H_j_* by removing the links from *j* to all other household members in *H_j_*, and delete the link from each of the other household members in *H_j_* to *j*.

### 4.2.4 Partnership Search Event

For each single (for example, male) individual *j* whose age is over 18, there is a chance for the individual to search (with an annual partnership searching rate) another single female individual *i*, whose age is over 18, as a partner to live together and form a new 2-member household. When this occurs, the household links for the simulated new couple consisting of individuals *j* and *i* are updated.

### 4.2.5 School Event

At the beginning of the simulation, the school event assigns all individuals with school ages between 6 and 12 or 13 and 18 years into primary schools or middle schools according to the observed school size data of Gansu province in China in 1975. Students with the same school ID are linked to each other in order to generate school links for each student *j* to all other schoolmates *Sch_j_*. During the simulation, the school event annually updates all individuals with school ages using the same method and school size data of the current year in order to reflect the changes in school student density from 1976 to 2009. This event always occurs each year until the end of the simulation.

## 4.3 Epidemic Events

### 4.3.1 Exogenous Infection Event

An exogenous infection event randomly chooses *K* individuals and lets them be infectious to spread the disease by calling the “Become Infectious Event” (see 4.3.2). At the beginning of the simulation, exogenous infections can start the epidemic in the totally susceptible population without mass immunity. During the simulation, this event occurs on day *θ* each year to introduce influenza every winter , because we assume that the simulated population is in Gansu province in northwestern China where the peak time of influenza season is in the winter.

### 4.3.2 Become Infectious Event

Once an individual *j* becomes infectious, an infectious period will be randomly generated (see Section 4.3.3). And during *j*’s infectious period, the contact times between he/she and each of his/her household members in the household links *H_j_* are stochastically scheduled using the exponential distribution with the contact rate between two household members per day, *contact_house_*=10, the transmission opportunity events for the infectious *j* and the susceptible household contacts will be active at the scheduled contact times. Similarly, during *j*’s infectious period the contact times between the infectious *j* and his/her casual contacts are randomly picked from the entire population and scheduled by an exponential distribution with casual contact rate per day, *contact_casual_*=16, and the transmission opportunity events for the infectious *j* and the susceptible casual contacts will be active at the scheduled times. For schoolmates of infectious *j*, during *j*’s infectious period the contact times between the *j* and his/her school contacts are randomly picked from *j*’s schoolmates *Sch_j_* and scheduled by an exponential distribution with school contact rate per day, *contact_school_* =10, and the transmission opportunity events for the infectious *j* and the susceptible school contacts will be active at the scheduled times.

### 4.3.3 Progression Event

For each individual *j* in the population who is infected (*Y_j_*=1), a progression event *P_j_* is active. The time that must elapse before the next progression event occurs is determined by sampling from an exponential distribution with a specified duration with minimum and maximum (depending on the stage and severity, as indicated below).

For all two severity types, the duration between *E*_1_ and *I*_1_,and the duration between *E’*_1_ and *I’*_1_ (the latent period) is chosen from the range 0.5-3 days; the duration between *I*_1_ and *I*_2_, and the duration between *I’*_1_ to *R* (asymptomatic period) is in the range of 0.5 to 2 days. For a non-mild case the symptomatic infectious period between *I*_2_ to *R* is 1 to 5 days. The non-mild case probability for an individual is in the range of 0.5 to 0.8.

Once an individual’s status has been updated, but the individual is still infectious (*I*_1_ and *I*_2_ for non-mild case, *I’*_1_ for mild case), the individual will have the potential to spread influenza via his/her contact networks (inside and outside of household, and school if *j* has schoolmates) by calling Become Infectious Event (described above) which will change the infection status of individual *j* to be infectious, and schedule contact times between the infectious *j* and contacts in his/her social networks, and then call Transmission Opportunity Events (see Section 4.3.4) to decide whether or not the transmission will occur between *j* and one of his/her contacts.

### 4.3.4 Transmission Opportunity Event

In the transmission opportunity event, the infectious individual *j* is able to transmit influenza to the individual *i* who is susceptible. This opportunity is true if the random number (uniform distribution between 0 and 1) is less than the product of the seasonal transmission probability ($P_{base}+\varepsilon\cos(2\pi(1+\frac{t-\theta}{\delta}))$) and the chance of escaping *i*’s current immunity ($1-M_{i}(t)$), which is the transmission probability of current contact $P_{trans}(t)=(P_{base}+\varepsilon\cos(2\pi(1+\frac{t-\theta}{\delta})))(1-M_{i}(t))$, where *P_base_* is the baseline transmission probability, *ε* (*-P_base_* < *ε* < *P_base_*) characterizes the degree of seasonality, *δ* denotes the total time of an epidemic season, *δ =* 365 days, *θ* is the peak time of an epidemic season (in northern China the peak time occurs in the winter on the 318th day of each year) , and *M_i_*(*t*) is the immunity level of the individual *i* (Equation 1). If this transmission is true, individual *i* becomes infected (*Y_i_* = 1) and its progression event is active to start the infection.

# 5 Uncertainty and Sensitivity Analysis

## 5.1 Calibration

We use Approximate Bayesian Computation method to optimize the parameters used in the model. We have described how we used this method and what parameters we calibrated in the main text (please see the *Calibration* section and Table 2 in the main text).

## 5.2 Partial Rank Correlation

We use the partial rank correlation coefficient as a measure of the sensitivity of any model output with respect to any parameter, holding all other parameters constant. The (sample) partial correlation coefficient of variables $W_{1}$ and $W_{2}$ with the collection $X_{1},\ldots,X_{N}$held constant (with respect to $X_{1},\ldots,X_{N}$) is defined to be the correlation of the residual of $W_{1}$ and $W_{2}$ after fitting these to $X_{1},\ldots,X_{N}$ , i.e. where $\hat{W}_{i}=X(X^{T}X)^{-1}X^{T}W_{i}$ ,$X$ is the usual design matrix (*i* th column of $X$ is $X_{i}$), and cor(*U_1_*, *U_2_*) denotes the (Pearson) product-moment correlation between an y quantities *U_1_* and *U_2_*.

If $X_{j}^{1},\ldots X_{j}^{i},...,X_{j}^{n}$ are *n* observations for some variable *X_j_*, a permutation *k_i_* of 1, …, *n* is ranking of $X_{j}^{1}...,X_{j}^{n}$ (and *k_i_* is a rank of $X_{j}^{i}$) if all the inequalities are strict the rank is unique; otherwise, the rank is defined as the average of all the possible rank orders (i.e., the rank of “tied” observations is the average rank); for conciseness, rank(*X_j_*) denotes the vector of ranks for the observations *X_j_*($X_{j}^{1}...,X_{j}^{n}$). The partial rank correlation of *W_1_* and *W_2_* holding *X_1_*, …, *X_N_* constant is computed as the partial correlation coefficient of rank(*W_1_*) and rank(*W_2_*) with respect to rank(*X_1_*), …, rank(*X_N_*)) (i.e., holding rank(*X_1_*), …, rank(*X_N_*) constant). For the results of PRCC, please see Table 3 (in the main text).

**Figures**

Figure S1: Model Structure.

Figure S2. Progression of the model.
Given a time *t*, each individual in the model is in one state of $S$(susceptible),$E_{1}^{'}$(mild exposure), $E_{1}$(not mild exposure), $I_{1}^{'}$(mild asymptomatic infectiousness),$I_{1}$(not mild asymptomatic infectiousness), $I_{2}$(symptomatic infectiousness) and$R$(recovered with immunity), and the population’s inflow and outflow are represented by each individual’s age-specific death rate *d* and age-specific fertility rate *b*.

Figure S3. Demographic calibration.
(A) Population projection using census data and a Leslie matrix. Case 1: population trajectory based on static data of survival probability (*sv*) and maternity rate of female (*mf*) in 1975. Case 2: population trajectory based on dynamic data of *sv* and *mf* in 1975, 1982, 1990, 2000 and 2009. Case 3: population trajectory based on dynamic data of *sv* and *mf* in 1975, 1982, 1990 and 2000. Case 4: population trajectory based on dynamic data of *sv* and *mf* in 1975, 1982 and 1990. Case 5: population trajectory based on dynamic data of *sv* and *mf* in 1975 and 1982. Case 6: population trajectory based on dynamic data of *sv* and *mf* in 1982, 1990 and 2000. Case 7: population trajectory based on dynamic data of *sv* and *mf* in 1982, 1990, 2000 and 2009. * The trajectory from Song J, Yu J (1988) Population system control: Springer. Note: this population projection did not include influenza transmission. After the population calibration, the simulations for influenza transmission only included 10,000 initial population. (B) Age structure. The solid lines in blue, red, green, orange, and purple are the simulated proportions in age groups 0 to 4, 5 to 9, 10 to 25, 26 to 49, and 50+, respectively. The dotted lines in the same colors are the observed proportions of the five age categories from census data. (C) Average household size. The blue, green and red lines are simulated average household sizes of each year under three scenarios: one-child policy, strict one-child policy, and absence of one-child policy. With a one-child policy (the blue line), the simulated average household size decreased from 4.2 in 1979 to about 3.5 in 2009, which is similar to the change in average household sizes reported in the census data: 4.43 (in 1964), 4.42 (1982), 3.96 (1990) and 3.44 (2009).

**Tables**

Table S1: The estimated *AR* and *SAR*.

Table S2: Some articles of influenza in China.

**References**

1. Macal CM, North MJ (2010) Tutorial on agent-based modelling and simulation. Journal of Simulation 4: 151-162.

2. Epstein JM (2009) Modelling to contain pandemics. Nature 460: 687-687.

3. Eubank S (2005) Network based models of infectious disease spread. Japanese Journal of Infectious Diseases 58: S9-S13.

4. Eubank S, Guclu H, Kumar VSA, Marathe MV, Srinivasan A, et al. (2004) Modelling disease outbreaks in realistic urban social networks. Nature 429: 180-184.

5. Germann TC, Kadau K, Longini IM, Macken CA (2006) Mitigation strategies for pandemic influenza in the United States. Proceedings of the National Academy of Sciences of the United States of America 103: 5935-5940.

6. Lee BY, Brown ST, Cooley PC, Zimmerman RK, Wheaton WD, et al. (2010) A Computer Simulation of Employee Vaccination to Mitigate an Influenza Epidemic. American Journal of Preventive Medicine 38: 247-257.

7. Jennings NR (1999) Agent-oriented software engineering. Multiple Approaches to Intelligent Systems, Proceedings 1611: 4-10.

8. Jennings NR (2000) On agent-based software engineering. Artificial Intelligence 117: 277-296.

9. Glasserman P, Yao DD (1992) Monotonicity in generalized semi-Markov processes. Mathematics of Operations Research: 1-21.

10. Hay AJ, Gregory V, Douglas AR, Lin YP (2001) The evolution of human influenza viruses. Philosophical Transactions of the Royal Society of London Series B-Biological Sciences 356: 1861-1870.

11. Ginsberg J, Mohebbi MH, Patel RS, Brammer L, Smolinski MS, et al. (2009) Detecting influenza epidemics using search engine query data. Nature 457: 1012-U1014.

12. Han T, Marasco WA (2011) Structural basis of influenza virus neutralization. Year in Immunology 1217: 178-190.

13. Koelle K, Cobey S, Grenfell B, Pascual M (2006) Epochal evolution shapes the phylodynamics of interpandemic influenza A (H3N2) in humans. Science 314: 1898-1903.

14. Finkelman BS, Viboud C, Koelle K, Ferrari MJ, Bharti N, et al. (2007) Global Patterns in Seasonal Activity of Influenza A/H3N2, A/H1N1, and B from 1997 to 2005: Viral Coexistence and Latitudinal Gradients. Plos One 2.

15. Koelle K, Khatri P, Kamradt M, Kepler TB (2010) A two-tiered model for simulating the ecological and evolutionary dynamics of rapidly evolving viruses, with an application to influenza. Journal of the Royal Society Interface 7: 1257-1274.

16. Shu YL, Fang LQ, de Vlas SJ, Gao Y, Richardus JH, et al. (2010) Dual Seasonal Patterns for Influenza, China. Emerging Infectious Diseases 16: 725-726.

17. Toni T, Welch D, Strelkowa N, Ipsen A, Stumpf MPH (2009) Approximate Bayesian computation scheme for parameter inference and model selection in dynamical systems. Journal of the Royal Society Interface 6: 187-202.

18. Beaumont MA, Zhang WY, Balding DJ (2002) Approximate Bayesian computation in population genetics. Genetics 162: 2025-2035.

19. Iman RL, Helton JC, Campbell JE (1981) An Approach to Sensitivity Analysis of Computer-Models .1. Introduction, Input Variable Selection and Preliminary Variable Assessment. Journal of Quality Technology 13: 174-183.

20. Iman RL, Helton JC, Campbell JE (1981) An Approach to Sensitivity Analysis of Computer-Models .2. Ranking of Input Variables, Response-Surface Validation, Distribution Effect and Technique Synopsis. Journal of Quality Technology 13: 232-240.

21. Blower SM, Dowlatabadi H (1994) Sensitivity and Uncertainty Analysis of Complex-Models of Disease Transmission - an Hiv Model, as an Example. International Statistical Review 62: 229-243.

22. Porco TC, Holbrook KA, Fernyak SE, Portnoy DL, Reiter R, et al. (2004) Logistics of community smallpox control through contact tracing and ring vaccination: a stochastic network model. Bmc Public Health 4.

23. Porco TC, Lewis B, Marseille E, Grinsdale J, Flood JM, et al. (2006) Cost-effectiveness of tuberculosis evaluation and treatment of newly-arrived immigrants. Bmc Public Health 6.
