# Supplementary material for: Effect of the One-Child Policy on Influenza Transmission in China: A Stochastic Transmission Model
Source: PLoS One. 2014 Feb 6;9(2):e84961. doi: 10.1371/journal.pone.0084961 (PMC3916292; doi:10.1371/journal.pone.0084961)
Supplement: Table S1 — The estimated AR and SAR . (DOC) [file pone.0084961.s005.doc]

Table S1: The estimated *AR* and *SAR*.

| AR References | Where | When | Estimates | Intervention? |
| --- | --- | --- | --- | --- |
| [1] | Franc and UK | 1984 to 2003 | 0.1 to 0.2 | Yes |
| [2] | Global | Average epidemics from 1918 to 1995 | 0.1 to 0.2 | Yes |
| [3] | US | 2003 | 0.09 to 0.20 | Yes |
| [4] | US | 1968 to 1998 | 0.1 to 0.2 | Yes |
| SAR References |  |  |  |  |
| [5] | France | 2000 | 0.18 | Yes |
| [6] | Seattle, US | 2000 to 2001 | 0.2 | Yes |
| [7] | Hong Kong | 2009 | 0.09 | Yes |
| [8] | Global | 1918 | 0.24 | Yes |
| [9] | US | 2009 | 0.1 to 0.4 | Yes |

[1] Truscott J, Fraser C, Cauchemez S, Meeyai A, Hinsley W, et al. (2012) Essential epidemiological mechanisms underpinning the transmission dynamics of seasonal influenza. Journal of the Royal Society Interface 9: 304-312.

[2] Cox NJ, Subbarao K (2000) Global epidemiology of influenza: Past and present. Annual Review of Medicine 51: 407-421.

[3] Molinari NAM, Ortega-Sanchez IR, Messonnier ML, Thompson WW, Wortley PM, et al. (2007) The annual impact of seasonal influenza in the US: Measuring disease burden and costs. Vaccine 25: 5086-5096.

[4] Koelle K, Cobey S, Grenfell B, Pascual M (2006) Epochal evolution shapes the phylodynamics of interpandemic influenza A (H3N2) in humans. Science 314: 1898-1903

[5] Viboud C, Boelle PY, Cauchemez S, Lavenu A, Valleron AJ, et al. (2004) Risk factors of influenza transmission in households. British Journal of General Practice 54: 684-689.

[6] Cannell JJ, Zasloff M, Garland CF, Scragg R, Giovannucci E (2008) On the epidemiology of influenza. Virology Journal 5.

[7] Cowling BJ, Chan KH, Fang VJ, Lau LLH, So HC, et al. (2010) Comparative Epidemiology of Pandemic and Seasonal Influenza A in Households. New England Journal of Medicine 362: 2175-2184.

[8] Fraser C, Cummings DA, Klinkenberg D, Burke DS, Ferguson NM (2011) Influenza transmission in households during the 1918 pandemic. American Journal of Epidemiology 174: 505-514.

[9] Cauchemez S, Donnelly CA, Reed C, Ghani AC, Fraser C, et al. (2009) Household transmission of 2009 pandemic influenza A (H1N1) virus in the United States. N Engl J Med 361: 2619-2627.
