# Supplementary material for: Effect of the One-Child Policy on Influenza Transmission in China: A Stochastic Transmission Model
Source: PLoS One. 2014 Feb 6;9(2):e84961. doi: 10.1371/journal.pone.0084961 (PMC3916292; doi:10.1371/journal.pone.0084961)
Supplement: Table S2 — Some articles of influenza in China. (DOC) [file pone.0084961.s006.doc]

***Table S2: Some articles of influenza in China.***

| ***Feature*** | ***Time and place*** | ***Risk group*** | ***Sampling or methods*** | ***Reference*** |
| --- | --- | --- | --- | --- |
| ***Influenza like illness*** | *2006-2010,Western Pacific Region;*  *2009-2010, Taiwan;*  *2007,Guangzhou, southern China;*  *2005-2008, Tianjin, northern China;*  *2009-2010, Nanjing, eastern China;*  *2004-2010, Hong Kong;*  *2006-2009,China;*  *2005-2009, Hong Kong;*  *2009, Guangzhou, southern China;*  *2004-2011, Hong Kong;*  *2008-2010, Wuhan, central China;*  *1998 January-2005 January,*  *Hong Kong;*  *2006-2009, nationwide China;*  *2006-2009, Shenzhen, southern China;*  *2009 June-2010 April, Beijing, China;*  *2010 June-2011 May, Beijing, China;*  *2007-2008, Beijing, China;*  *1991-2000, Guangdong, southern China;*  *2008 January-April, Shanghai, eastern China;*  *2009, Hangzhou, eastern China;*  *2009, Qingdao, eastern China;*  *2000-2007, Gansu, northwestern China;*  *2003 December-2005 January, Tianjin, northern China;*  *2005 October-2010 February, Jiangsu, eastern China;*  *2011 January-December, Gansu, northwest China.* | *All ages;*  *all ages;*  *age-specific;*  *all ages;*  *pregnant women;*  *all ages;*  *all ages;*  *all ages;*  *all ages;*  *all ages;*  *children;*  *all ages;*  *all ages;*  *all ages;*  *all ages;*  *adult patients (age >16);*  *all ages;*  *all ages;*  *ages: 7 to 75;*  *all ages;*  *all ages;*  *all ages;*  *all ages;*  *all age;*  *children.* | *Email survey, questionnaire;*  *patients visited Emergency Room, PCR;*  *population-based household survey;*  *surveillance from sentinel hospitals;*  *serum samples of unvaccinated pregnant women;*  *sentinel hospitals and general practitioners;*  *197 sentinel hospitals;*  *41 public hospitals;*  *confirmed case reported by local hospitals*  *specimens provided by Center for Health Protection;*  *children hospital of Wuhan;*  *weekely ILI case was reported by local sentinel practitioners;*  *surveillance data from China CDC;*  *loclal flu surveillance system;*  *local surveillance systematic;*  *a total of 279 throat swabs were by local hospital;*  *influenza Surveillance System in Beijing;*  *data were collected from the influenza surveillance network in Guangdong;*  *thraot swabs were collected from 365 ILI patients;*  *local flu surveillance system;*  *sample collection was did by three local sentinel hospitals;*  *surveillance data from local CDC;*  *surveillance data from local CDC;*  *the number of weekly ILI cases;*  *surveillance data from local CDC.* | *[null & System 2012];*  *[Chuang et al. 2012] ;*  *[Guo et al. 2011] ;*  *[Hu,Quirchmayr,Winiwarter & Cui 2012];*  *[Huo et al. 2011] ;*  *[Lau,Cheng,Ip & Cowling 2012];*  *[Liang et al. 2012] ;*  *[Yang et al. 2012a] ;*  *[Yang et al. 2012b] ;*  *[Mak,Wong,Ho & Lim 2012] ;*  *[Peng et al. 2012] ;*  *[Cowling,Wong,Ho,Riley & Leung 2006];*  *[Shu,Fang,de Vlas,Gao,Richardus & Cao 2010];*  *[Wang et al. 2011a];*  *[Yang,Qian,Peng,Liang,Huang & Wang 2010];*  *[Yang et al. 2012c];*  *[Yang et al. 2009];*  *[Huang,Ni,Shen,Zhou,Peng & Liu 2001];*  *[Ju et al. 2010];*  *[Liu et al. 2010a];*  *[Wang et al. 2011b];*  *[Li,Yu,Chen & Liu 2008];*  *[xiu Wang,lan Shan,Gao & Li 2005];*  *[Zhu et al. 2011];*  *[Huang et al. 2013].* |
| ***Secondary attack rate in household*** | *2008, Hong Kong;*  *2008-2011, Guangzhou, southern China;*  *2009 August-October, Guangzhou, southern China;*  *2009 May-October, Beijing, northern China;*  *2009 April-October, Hong Kong;*  *2009, worldwide;*  *2009 May-October; Beijing, northern China;*  *2009, Shenzhen, southern China;*  *2009 July-August, Hong Kong;*  *2009 July, Dongguan, southern China;*  *2009, Henan, middle China;*  *2011-2012, Hong Kong;*  *2010, Zhuhai, southern China.* | *259 households;*  *school, kindergarten & groups with outbreaks;*  *students in boarding schools;*  *close contacts of index cases;*  *117 households;*  *all ages;*  *close contacts of index cases;*  *all ages;*  *all ages;*  *students in local primary school;*  *students and employees in a middle school;*  *adults in a hospital;*  *all ages.* | *Community based study;*  *confirmed cases reported to local CDC;*  *lab confirmed cases;*  *close contact tracing from 613 index cases with pH1N1;*  *a randomized trial from 119 families;*  *a systematic Review;*  *close contact tracing from 613 index cases with pH1N1;*  *2063 lab confirme case from local flu surveillance systematic;*  *348 index patients with acute respiratory illness from 14 outpatient clinics in Hong Kong;*  *retrospective case finding by reviewing*  the school’s absentee log and retrieving medical records*;*  *a retrospective cohort study was conducted among all students and employees in a local middle school by a telephone survey and laboratory inspection;*  *an outbreak investigation was performed for a cluster of influenza cases which occurred in an adult psychiatric ward of a general hospital in November 2011;*  *outpatients who sought attention in the Outpatient Department of Zhuhai Municipal People’s Hospital in 2010.* | *[Klick,Leung & Cowling 2012];*  *[Li et al. 2011a];*  *[Li et al. 2011b];*  *[Pang et al. 2011];*  *[Klick et al. 2011];*  *[Lau,Nishiura,Kelly,Ip,Leung & Cowling 2012];*  *[Pang et al. 2011];*  *[Xie et al. 2012];*  *[Cowling et al. 2010];*  *[Huai et al. 2010];*  *[Wang et al. 2012];*  *[Chan et al. 2013];*  *[Li,Wei,Tan & Wang 2013].* |
| ***Annual attack rate*** | *2009 May-September, China main land;*  *2009 May-September, Hong Kong;*  *2009 June, A Train in China;*  *2009-2010, Hong Kong;*  *2009 April-December, Hong Kong;*  *2009, Hong Kong;*  *2009 May-2010 January, Henan, meddle China.* | *All ages;*  *4 age groups;*  *all ages of passengers;*  *all ages*  *all ages;*  *all ages;*  *all ages.* | *Confirmed cases reported to China Information System for Disease Control and Prevention;*  *lab confirmed cases;*  *lab confirmed cases;*  *a cohort of households in Hong Kong*  *serum specimens from blood donors, hospital outpatients, and community pediatric cohort study;*  *14766 collected serum specimens during the first wave of 2009 pandemic in Hong Kong;*  *3768 samples were tested by local CDC.* | *[Fang et al. 2012] ;*  *[Lee & Wong 2010];*  *[Cui et al. 2011];*  *[Riley et al. 2011];*  *[Wu et al. 2010a];*  *[Wu et al. 2011a];*  *[Chang,Wang,Zhou,Chang,Li & Shi 2012].* |
| ***Risk of seroconversion*** | *2009-2010, Nanjing, eastern China;*  *2010 May, Jiangsu, eastern China;*  *2009 July-September, Guangdong, southern China;*  *2009, China;*  *2010 January, Beijing, northern China;*  *2009-2010, Beijing, northern China;*  *2009-2010, Guangdong, southern China;*  *2009-2010, Guangdong, southern China;*  *2010 February-March, Hong Kong;*  *2010 January, Mainland China;*  *2009, Beijing, northern China;*  *2009, Guangxi, southwestern China;*  *1977, Beijing, southern China;*  *2009 May-June, Hong Kong;*  *1918, 1968, China*  *2009, China;*  *2009, Gansu, northwestern China.* | *Pregnant women;*  *people from rural areas;*  *all ages;*  *cohort study;*  *all ages;*  *all ages;*  *all ages;*  *age from 6 to 59 months;*  *health care workers;*  *all ages;*  *all ages;*  *all ages;*  *all ages;*  *all ages;*  *all ages.* | *Serum samples of unvaccinated pregnant women;*  *serum samples of unvaccinated people from rural areas;*  *serum samples from 151 local residents;*  *a cohort of 773 individuals who received a monovalent vaccine;*  *4601 participants from a random serological survey;*    *three serological surveys in Beijing;*  *three serological surveys in Guangdong;*  *three serological surveys in Guangdong;*  *a cross-sectional study among health care workers in Hong Kong;*  *50111 serum samples were collected using a multistage-stratified random sampling method;*  *a serological survey in 710 residents of Beijing;*  *4043 unvaccinated and 22 vaccinated subjects in Guangxi;*  *serum samples were collected from 156 resident in Beijing;*  *laboratory confirmed cases;*  *Simulated results by a Monte-Carlo model;*  *“These results suggest there are characteristics of communities that drive influenza transmission dynamics apart from individual and household level risk factors, and that such factors have effects independent of strain.”*  *surveillance data from local CDC.* | *[Huo et al. 2012];*  *[Chen et al. 2011];*  *[Lessler et al. 2012];*  *[Liu et al. 2010b];*  *[Tian et al. 2011];*  *[Yang et al. 2011];*  *[Yang et al. 2012d];*  *[Yang,Dong & Fu 2012e];*  *[Zhou et al. 2011];*  *[Xu et al. 2011];*  *[Deng et al. 2011];*  *[Chen et al. 2009];*  *[Kung,Jen,Yuan,Tien & Chu 1978];*  *[Mak,Choy,Lee,Wong,Ng & Lim 2010];*  *[Yu,Feng,Peng,Feng,Shay & Yang 2009];*  *[Lessler et al. 2011];*  *[shan Yu,yu Li & ming Gao 2010].* |
| ***Time series*** | *2009 September-2010 January*  *2008, Hong Kong;*  *2007, Hong Kong;* | *Age 18- 35;*  *volunteers;*  *all ages.* | *37 patient with seasonal influenza;*  *Community-based study.* | *[Jia et al. 2011];*  *[Lau et al. 2010];*  *[Cowling,Fang,Riley,Peiris & Leung 2009].* |
| ***The Reproduction number*** | *2009 July-2010 March, Taiwan;*  *2009 June-November, China;*  *2009 June-2010 March, Fujian, southeastern China;*  *2009, Beijing, northern China;*  *2009, Hong Kong;*  *2009 May-November, northern China;*  *2009 June-2010 June, Zhejiang, eastern China;*  *2009 May-August, 6 countries in southern hemisphere;*  *2009, Hong Kong.* | *All ages;*  *all ages;*  *all ages;*  *all ages;*  *children;*  *children;*  *all ages;*  *all ages;* | *Confirmed cases and partial school closure*  *confirmed case reported by CDC*  *1414 cases reported by local CDC*  *10884 reported cases in Beijing;*  *children in kindergartens and primary schools;*  *nationwide flu surveillance system;*  *local flu surveillance system;*  *surveillance data collected from Argentina, Chile, Brazil, New Zealand, Australia, South Africa;*  *serological surveillance study in Hong Kong.* | *[Hsieh et al. 2011];*  *[Jin,Zhang,Song,Sun,Kan & Zhu 2011];*  *[Shen & Niu 2012];*  *[Wang et al. 2010];*  *[Wu et al. 2010b];*  *[Yu et al. 2012];*  *[Fu,Chen,Chen,Wang & Ling 2011];*  *[Hsieh 2010];*  *[Wu et al. 2011b].* |
| ***Travel Patten*** | *2007, northern and southern China;*  *2009 July-August.* |  | *Difference in travel distance for the population living in developed area and rural area;*  *53 suspected cases and 67 close contacts provided by Chinese Ministry of Health.* | *[Garske et al. 2011];*  *[Liu et al. 2010c].* |
| ***Seasonality*** | *1993-1997, China;* | *All ages.* | *Influenza surveillance in China;* | *[Hampson 1999] .* |
| ***Review articles*** | *The birthplace of modern influenza pandemics seems to be China.* |  |  | *[Laver & Garman 2002].* |
| ***Rate of Hospitalization*** | *1996-2000, Hong Kong.* | *All ages.* | *Weekly data from 14 hospitals in Hong Kong from 1996 to 2000.* | *[Wong,Yang,Chan,Chan,Hedley & Peiris 2009].* |
| ***Others:***  *Southern China is unlikely to represent an epicenter of global influenza activity;* *Heterogeneity in viral shedding among individuals;* *Risk factors among students.* | *1996-2009, Shengzhen, southern China;*  *2008-2009, Hong Kong;*  *2009 October-2010 January, Beijing, northern China.* | *All ages;*  *all ages;*  *students 6 to 19 years old.* | *epidemiological and virological data on influenza recorded over 15 years from local CDC;*  *two large community-based trials studying household transmission of influenza conducted in Hong Kong;*  *a case–control study (304 case-patients and 608 controls, age range 6–19 years) conducted in Beijing.* | *[Cheng et al. 2013];*  *[Lau et al. 2013];*  *[Zheng et al. 2013].* |

## References

**Chan, M. C. W.; Lee, N.; Ngai, K. L. K.; Wong, B. C. K.; Lee, M. K. P.; Choi, K. W.; Lai, R. W. M. and Chan, P. K. S.** (**2013**). *A "pre-seasonal" hospital outbreak of influenza pneumonia caused by the drift variant A/Victoria/361/2011-like H3N2 viruses, Hong Kong, 2011.*, J Clin Virol 56 : 219-225.

**Chang, C.; Wang, J.-W.; Zhou, X.-X.; Chang, Z.-J.; Li, X.-T. and Shi, T.-L.** (**2012**). *[Evaluating the current protocol of influenza A (H1N1) based on the epidemic situations of Zhengzhou,a middle-sized city in China].*, Zhonghua Shi Yan He Lin Chuang Bing Du Xue Za Zhi 26 : 54-56.

**Chen, H.; Wang, Y.; Liu, W.; Zhang, J.; Dong, B.; Fan, X.; de Jong, M. D.; Farrar, J.; Riley, S.; Smith, G. J. D. and Guan, Y.** (**2009**). *Serologic survey of pandemic (H1N1) 2009 virus, Guangxi Province, China.*, Emerg Infect Dis 15 : 1849-1850.

**Chen, Y.; Zheng, Q.; Yang, K.; Zeng, F.; Lau, S.-Y.; Wu, W. L.; Huang, S.; Zhang, J.; Chen, H. and Xia, N.** (**2011**). *Serological survey of antibodies to influenza A viruses in a group of people without a history of influenza vaccination.*, Clin Microbiol Infect 17 : 1347-1349.

**Cheng, X.; Tan, Y.; He, M.; Lam, T. T.-Y.; Lu, X.; Viboud, C.; He, J.; Zhang, S.; Lu, J.; Wu, C.; Fang, S.; Wang, X.; Xie, X.; Ma, H.; Nelson, M. I.; fu Kung, H.; Holmes, E. C. and Cheng, J.** (**2013**). *Epidemiological dynamics and phylogeography of influenza virus in southern China.*, J Infect Dis 207 : 106-114.

**Chuang, J.-H.; Huang, A. S.; Huang, W.-T.; Liu, M.-T.; Chou, J.-H.; Chang, F.-Y. and Chiu, W.-T.** (**2012**). *Nationwide surveillance of influenza during the pandemic (2009-10) and post-pandemic (2010-11) periods in Taiwan.*, PLoS One 7 : e36120.

**Cowling, B. J.; Chan, K. H.; Fang, V. J.; Lau, L. L. H.; So, H. C.; Fung, R. O. P.; Ma, E. S. K.; Kwong, A. S. K.; Chan, C.-W.; Tsui, W. W. S.; Ngai, H.-Y.; Chu, D. W. S.; Lee, P. W. Y.; Chiu, M.-C.; Leung, G. M. and Peiris, J. S. M.** (**2010**). *Comparative epidemiology of pandemic and seasonal influenza A in households.*, N Engl J Med 362 : 2175-2184.

**Cowling, B. J.; Fang, V. J.; Riley, S.; Peiris, J. S. M. and Leung, G. M.** (**2009**). *Estimation of the serial interval of influenza.*, Epidemiology 20 : 344-347.

**Cowling, B. J.; Wong, I. O. L.; Ho, L.-M.; Riley, S. and Leung, G. M.** (**2006**). *Methods for monitoring influenza surveillance data.*, Int J Epidemiol 35 : 1314-1321.

**Cui, F.; Luo, H.; Zhou, L.; Yin, D.; Zheng, C.; Wang, D.; Gong, J.; Fang, G.; He, J.; McFarland, J. and Yu, H.** (**2011**). *Transmission of pandemic influenza A (H1N1) virus in a train in China.*, J Epidemiol 21 : 271-277.

**Deng, Y.; Pang, X. H.; Yang, P.; Shi, W. X.; Tian, L. L.; Liu, B. W.; Li, S.; Cui, S. J.; Li, Y.; Lu, G. L.; Zhang, L.; Zhang, X.; Liu, B.; Seale, H.; Huang, F. and Wang, Q. Y.** (**2011**). *Serological survey of 2009 H1N1 influenza in residents of Beijing, China.*, Epidemiol Infect 139 : 52-58.

**Fang, L.-Q.; Wang, L.-P.; de Vlas, S. J.; Liang, S.; Tong, S.-L.; Li, Y.-L.; Li, Y.-P.; Qian, Q.; Yang, H.; Zhou, M.-G.; Wang, X.-F.; Richardus, J. H.; Ma, J.-Q. and Cao, W.-C.** (**2012**). *Distribution and risk factors of 2009 pandemic influenza A (H1N1) in mainland China.*, Am J Epidemiol 175 : 890-897.

**Fu, J.; Chen, S.; Chen, J.; Wang, J. and Ling, C.** (**2011**). *Epidemiological characteristics of pandemic influenza A (H1N1-2009) in Zhanjiang, China.*, Pan Afr Med J 10 : 54.

**Garske, T.; Yu, H.; Peng, Z.; Ye, M.; Zhou, H.; Cheng, X.; Wu, J. and Ferguson, N.** (**2011**). *Travel patterns in China.*, PLoS One 6 : e16364.

**Guo, R. N.; Zheng, H. Z.; Li, J. S.; Sun, L. M.; Li, L. H.; Lin, J. Y. and He, J. F.** (**2011**). *A population-based study on incidence and economic burden of influenza-like illness in south China, 2007.*, Public Health 125 : 389-395.

**Hampson, A. W.** (**1999**). *Epidemiological data on influenza in Asian countries.*, Vaccine 17 Suppl 1 : S19-S23.

**Hsieh, Y.-H.** (**2010**). *Pandemic influenza A (H1N1) during winter influenza season in the southern hemisphere.*, Influenza Other Respi Viruses 4 : 187-197.

**Hsieh, Y.-H.; Cheng, K.-F.; Wu, T.-N.; Li, T.-C.; Chen, C.-Y.; Chen, J.-H.; Lin, M.-H.; null, C. f. I. E. and Team, R.** (**2011**). *Transmissibility and temporal changes of 2009 pH1N1 pandemic during summer and fall/winter waves.*, BMC Infect Dis 11 : 332.

**Huai, Y.; Lin, J.; Varma, J. K.; Peng, Z.; He, J.; Cheng, C.; Zhong, H.; Chen, Y.; Zheng, Y.; Luo, Y.; Liang, W.; Wu, X.; Huang, Z.; McFarland, J.; Feng, Z.; Uyeki, T. M. and Yu, H.** (**2010**). *A primary school outbreak of pandemic 2009 influenza A (H1N1) in China.*, Influenza Other Respi Viruses 4 : 259-266.

**Huang, G.; Yu, D.; Mao, N.; Zhu, Z.; Zhang, H.; Jiang, Z.; Li, H.; Zhang, Y.; Shi, J.; Zhang, S.; Wang, X. and Xu, W.** (**2013**). *Viral etiology of acute respiratory infection in gansu province, china, 2011.*, PLoS One 8 : e64254.

**Huang, P.; Ni, H.; Shen, G.; Zhou, H.; Peng, G. and Liu, S.** (**2001**). *Analysis of the 1991-2000 influenza epidemic in Guangdong Province, China.*, Southeast Asian J Trop Med Public Health 32 : 787-790.

**Hu, X.-Q.; Quirchmayr, G.; Winiwarter, W. and Cui, M.** (**2012**). *Influenza early warning model based on Yunqi theory.*, Chin J Integr Med 18 : 192-196.

**Huo, X.; Qi, X.; Tang, F.; Zu, R.; Li, L.; Wu, B.; Qin, Y.; Ji, H.; Fu, J.; Wang, S.; Tian, H.; Hu, Z.; Yang, H.; Zhou, M.; Wang, H. and Zhu, F.** (**2011**). *Seroprevalence of pandemic (H1N1) 2009 in pregnant women in China: an observational study.*, PLoS One 6 : e17995.

**Huo, X.; Zu, R.; Qi, X.; Qin, Y.; Li, L.; Tang, F.; Hu, Z. and Zhu, F.** (**2012**). *Seroprevalence of avian influenza A (H5N1) virus among poultry workers in Jiangsu Province, China: an observational study.*, BMC Infect Dis 12 : 93.

**Jia, N.; Gao, Y.; Suo, J.-J.; Xie, L.-J.; Yan, Z.-Q.; Xing, Y.-B.; He, L. and Liu, Y.-X.** (**2011**). *Viral shedding in Chinese young adults with mild 2009 H1N1 influenza.*, Chin Med J (Engl) 124 : 1576-1579.

**Jin, Z.; Zhang, J.; Song, L.-P.; Sun, G.-Q.; Kan, J. and Zhu, H.** (**2011**). *Modelling and analysis of influenza A (H1N1) on networks.*, BMC Public Health 11 Suppl 1 : S9.

**Ju, L.; Jiang, L.; Yang, J.; Shi, Q.; Jiang, Q.; Shen, H.; Tan, Y. and Lu, Y.** (**2010**). *Co-infection with influenza A/H1N1 and A/H3N2 viruses in a patient with influenza-like illness during the winter/spring of 2008 in Shanghai, China.*, J Med Virol 82 : 1299-1305.

**Klick, B.; Nishiura, H.; Ng, S.; Fang, V. J.; Leung, G. M.; Peiris, J. S. M. and Cowling, B. J.** (**2011**). *Transmissibility of seasonal and pandemic influenza in a cohort of households in Hong Kong in 2009.*, Epidemiology 22 : 793-796.

**Klick, B.; Leung, G. M. and Cowling, B. J.** (**2012**). *Optimal design of studies of influenza transmission in households. I: case-ascertained studies.*, Epidemiol Infect 140 : 106-114.

**Kung, H. C.; Jen, K. F.; Yuan, W. C.; Tien, S. F. and Chu, C. M.** (**1978**). *Influenza in China in 1977: recurrence of influenzavirus A subtype H1N1.*, Bull World Health Organ 56 : 913-918.

**Lau, L. L. H.; Cowling, B. J.; Fang, V. J.; Chan, K.-H.; Lau, E. H. Y.; Lipsitch, M.; Cheng, C. K. Y.; Houck, P. M.; Uyeki, T. M.; Peiris, J. S. M. and Leung, G. M.** (**2010**). *Viral shedding and clinical illness in naturally acquired influenza virus infections.*, J Infect Dis 201 : 1509-1516.

**Lau, L. L. H.; Nishiura, H.; Kelly, H.; Ip, D. K. M.; Leung, G. M. and Cowling, B. J.** (**2012**). *Household transmission of 2009 pandemic influenza A (H1N1): a systematic review and meta-analysis.*, Epidemiology 23 : 531-542.

**Lau, L. L. H.; Ip, D. K. M.; Nishiura, H.; Fang, V. J.; Chan, K.-H.; Peiris, J. S. M.; Leung, G. M. and Cowling, B. J.** (**2013**). *Heterogeneity in viral shedding among individuals with medically attended influenza A virus infection.*, J Infect Dis 207 : 1281-1285.

**Lau, E. H. Y.; Cheng, C. K. Y.; Ip, D. K. M. and Cowling, B. J.** (**2012**). *Situational awareness of influenza activity based on multiple streams of surveillance data using multivariate dynamic linear model.*, PLoS One 7 : e38346.

**Laver, G. and Garman, E.** (**2002**). *Pandemic influenza: its origin and control.*, Microbes Infect 4 : 1309-1316.

**Lee, S. S. and Wong, N. S.** (**2010**). *Reconstruction of epidemic curves for pandemic influenza A (H1N1) 2009 at city and sub-city levels.*, Virol J 7 : 321.

**Lessler, J.; Cummings, D. A. T.; Read, J. M.; Wang, S.; Zhu, H.; Smith, G. J. D.; Guan, Y.; Jiang, C. Q. and Riley, S.** (**2011**). *Location-specific patterns of exposure to recent pre-pandemic strains of influenza A in southern China.*, Nat Commun 2 : 423.

**Lessler, J.; Riley, S.; Read, J. M.; Wang, S.; Zhu, H.; Smith, G. J. D.; Guan, Y.; Jiang, C. Q. and Cummings, D. A. T.** (**2012**). *Evidence for antigenic seniority in influenza A (H3N2) antibody responses in southern China.*, PLoS Pathog 8 : e1002802.

**Li, H.-Y.; Yu, D.-S.; Chen, J.-H. and Liu, X.-Z.** (**2008**). *[Analysis of influenza surveillance from 2000 to 2007 in Gansu province].*, Zhonghua Shi Yan He Lin Chuang Bing Du Xue Za Zhi 22 : 266-268.

**Li, H.; Wei, Q.; Tan, A. and Wang, L.** (**2013**). *Epidemiological analysis of respiratory viral etiology for influenza-like illness during 2010 in Zhuhai, China.*, Virol J 10 : 143.

**Li, T.; Fu, C.; Di, B.; Wu, J.; Yang, Z.; Wang, Y.; Li, M.; Lu, J.; Chen, Y.; Lu, E.; Geng, J.; Hu, W.; Dong, Z.; Li, M.-F.; Zheng, B.-J.; Cao, K.-Y. and Wang, M.** (**2011a**). *A two-year surveillance of 2009 pandemic influenza A (H1N1) in Guangzhou, China: from pandemic to seasonal influenza?*, PLoS One 6 : e28027.

**Li, T.; Liu, Y.; Di, B.; Wang, M.; Shen, J.; Zhang, Y.; Chen, X.; Yuan, J.; Wu, J.; Li, K.; Lu, E.; Wu, Y.; Hao, A.; Chen, X.; Wang, Y.; Liu, J.; Pickerill, S. and Zheng, B.** (**2011b**). *Epidemiological investigation of an outbreak of pandemic influenza A (H1N1) 2009 in a boarding school: serological analysis of 1570 cases.*, J Clin Virol 50 : 235-239.

**Liang, W.; Feng, L.; Xu, C.; Xiang, N.; Zhang, Y.; Shu, Y.; Wang, H.; Luo, H.; Yu, H.; Liang, X.; Li, D.; Lee, C.-K.; Feng, Z.; Hou, Y.; Wang, Y.; Chen, Z. and Yang, W.** (**2012**). *Response to the first wave of pandemic (H1N1) 2009: experiences and lessons learnt from China.*, Public Health 126 : 427-436.

**Liu, S.-L.; Zhang, Z.-R.; Wang, C.; Dong, Y.; Cui, L.-B.; Yang, X.-H.; Sun, Z.; Wang, J.; Chen, J.; Huang, R.-J.; Miao, F.; Ruan, B.; Xie, L.; He, H.-X. and Deng, J.** (**2010a**). *2009 pandemic characteristics and controlling experiences of influenza H1N1 virus 1 year after the inception in Hangzhou, China.*, J Med Virol 82 : 1985-1995.

**Liu, W.; de Vlas, S. J.; Tang, F.; Ma, M.-J.; Wei, M.-T.; Liu, L.-J.; Li, Z.-D.; Zhang, L.; Xin, Z.-T.; Tong, Y.-G.; Jiang, T.; Zhang, X.-A.; He, C.; Li, C.; Xu, X.-N.; Yang, H.; Richardus, J. H. and Cao, W.-C.** (**2010b**). *Clinical and immunological characteristics of patients with 2009 pandemic influenza A (H1N1) virus infection after vaccination.*, Clin Infect Dis 51 : 1028-1032.

**Liu, W.; Jiang, T.; Li, X.-F.; Tang, F.; Wei, M.-T.; Yu, M.; Zhao, H.; Yu, X.-D.; Liu, L.-J.; Qin, C.-F. and Cao, W.-C.** (**2010c**). *Community transmission of pandemic influenza A (H1N1) in China.*, Infect Control Hosp Epidemiol 31 : 961-963.

**Mak, G. C.; Choy, P. W. W.; Lee, W. Y.; Wong, A. H.; Ng, K. C. and Lim, W.** (**2010**). *Sero-immunity and serologic response to pandemic influenza A (H1N1) 2009 virus in Hong Kong.*, J Med Virol 82 : 1809-1815.

**Mak, G. C.; Wong, A. H.; Ho, W. Y. Y. and Lim, W.** (**2012**). *The impact of pandemic influenza A (H1N1) 2009 on the circulation of respiratory viruses 2009-2011.*, Influenza Other Respi Viruses 6 : e6-10.

**null, W. P. R. G. I. S. and System, R.** (**2012**). *Epidemiological and virological characteristics of influenza in the Western Pacific Region of the World Health Organization, 2006-2010.*, PLoS One 7 : e37568.

**Pang, X.-h.; Yang, P.; Li, S.; Zhang, L.; Tian, L.-l.; Li, Y.; Liu, B.; Zhang, Y.; Liu, B.-w.; Huang, R.-g.; Li, X.-y. and Wang, Q.-y.** (**2011**). *[Epidemiological characteristics of infection for close contacts of pandemic (H1N1) 2009 in Beijing]*, Zhonghua Jie He He Hu Xi Za Zhi 34 : 348-352.

**Peng, J.; Kong, W.; Guo, D.; Liu, M.; Wang, Y.; Zhu, H.; Pang, B.; Miao, X.; Yu, B.; Luo, T.; Hu, Q. and Zhou, D.** (**2012**). *The epidemiology and etiology of influenza-like illness in Chinese children from 2008 to 2010.*, J Med Virol 84 : 672-678.

**Pang, X.; Yang, P.; Li, S.; Zhang, L.; Tian, L.; Li, Y.; Liu, B.; Zhang, Y.; Liu, B.; Huang, R.; Li, X. and Wang, Q.** (**2011**). *Pandemic (H1N1) 2009 among quarantined close contacts, Beijing, People's Republic of China.*, Emerg Infect Dis 17 : 1824-1830

**Riley, S.; Kwok, K. O.; Wu, K. M.; Ning, D. Y.; Cowling, B. J.; Wu, J. T.; Ho, L.-M.; Tsang, T.; Lo, S.-V.; Chu, D. K. W.; Ma, E. S. K. and Peiris, J. S. M.** (**2011**). *Epidemiological characteristics of 2009 (H1N1) pandemic influenza based on paired sera from a longitudinal community cohort study.*, PLoS Med 8 : e1000442.

**Shen, J. and Niu, J.** (**2012**). *Epidemiologic parameters and evaluation of control measure for 2009 novel influenza a (H1N1) in Xiamen, Fujian Province, China.*, Virol J 9 : 20.

**Shu, Y.-L.; Fang, L.-Q.; de Vlas, S. J.; Gao, Y.; Richardus, J. H. and Cao, W.-C.** (**2010**). *Dual seasonal patterns for influenza, China.*, Emerg Infect Dis 16 : 725-726.

**Tian, L.-l.; Shi, W.-x.; Ying-Deng; Pang, X.-h.; Peng-Yang; Fang-Huang; Cui, S.-j.; Xin-Zhang; Zhang, D.-t. and Wang, Q.-y.** (**2011**). *Serologic survey of pandemic influenza A (H1N1 2009) in Beijing, China.*, Prev Med 52 : 71-74.

**Wang, C.; Yu, E.; Xu, B.; Wang, W.; Li, L.; Zhang, W.; Zhang, M. and Li, W.** (**2012**). *Epidemiological and clinical characteristics of the outbreak of 2009 pandemic influenza A (H1N1) at a middle school in Luoyang, China.*, Public Health 126 : 289-294.

**xiu Wang, J.; lan Shan, A.; Gao, L. and Li, L.** (**2005**). *[A study on the influenza surveillance program in Tianjin, 2004].*, Zhonghua Liu Xing Bing Xue Za Zhi 26 : 848-850.

**Wang, X.; Cheng, X. W.; Ma, H. W.; He, J. F.; Xie, X.; Fang, S. S.; Wu, C. L.; Lu, X.; Mei, S. J.; Li, Y. and Cheng, J. Q.** (**2011a**). *Influenza surveillance in Shenzhen, the largest migratory metropolitan city of China, 2006-2009.*, Epidemiol Infect 139 : 1551-1559.

**Wang, X.; Yang, P.; Seale, H.; Zhang, Y.; Deng, Y.; Pang, X.; He, X. and Wang, Q.** (**2010**). *Estimates of the true number of cases of pandemic (H1N1) 2009, Beijing, China.*, Emerg Infect Dis 16 : 1786-1788.

**Wang, Z.-G.; Yi, Y.; Yang, T.-T.; Liu, X.-L.; Jiang, F.-C.; Wang, Z.-Y. and Chen, J.-M.** (**2011b**). *Emergency surveillance of influenza during 2009 in the Chinese city of Qingdao.*, Influenza Other Respi Viruses 5 : 53-59.

**Wong, C. M.; Yang, L.; Chan, E.; Chan, K. H.; Hedley, A. J. and Peiris, J. S. M.** (**2009**). *Influenza-associated hospitalisation.*, Hong Kong Med J 15 Suppl 9 : 35-37.

**Wu, J. T.; Cowling, B. J.; Lau, E. H. Y.; Ip, D. K. M.; Ho, L.-M.; Tsang, T.; Chuang, S.-K.; Leung, P.-Y.; Lo, S.-V.; Liu, S.-H. and Riley, S.** (**2010b**). *School closure and mitigation of pandemic (H1N1) 2009, Hong Kong.*, Emerg Infect Dis 16 : 538-541.

**Wu, J. T.; Ho, A.; Ma, E. S. K.; Lee, C. K.; Chu, D. K. W.; Ho, P.-L.; Hung, I. F. N.; Ho, L. M.; Lin, C. K.; Tsang, T.; Lo, S.-V.; Lau, Y.-L.; Leung, G. M.; Cowling, B. J. and Peiris, J. S. M.** (**2011a**). *Estimating infection attack rates and severity in real time during an influenza pandemic: analysis of serial cross-sectional serologic surveillance data.*, PLoS Med 8 : e1001103.

**Wu, J. T.; Ma, E. S. K.; Lee, C. K.; Chu, D. K. W.; Ho, P.-L.; Shen, A. L.; Ho, A.; Hung, I. F. N.; Riley, S.; Ho, L. M.; Lin, C. K.; Tsang, T.; Lo, S.-V.; Lau, Y.-L.; Leung, G. M.; Cowling, B. J. and Peiris, J. S. M.** (**2010a**). *The infection attack rate and severity of 2009 pandemic H1N1 influenza in Hong Kong.*, Clin Infect Dis 51 : 1184-1191.

**Wu, J. T.; Ma, E. S. K.; Lee, C. K.; Chu, D. K. W.; Ho, P.-L.; Shen, A. L.; Ho, A.; Hung, I. F. N.; Riley, S.; Ho, L. M.; Lin, C. K.; Tsang, T.; Lo, S.-V.; Lau, Y.-L.; Leung, G. M.; Cowling, B. J. and Peiris, J. S. M.** (**2011b**). *A serial cross-sectional serologic survey of 2009 Pandemic (H1N1) in Hong Kong: implications for future pandemic influenza surveillance.*, Influenza Other Respi Viruses 5 Suppl 1 : 190-194.

**Xie, X.; Lu, S. Q. Y.; Cheng, J. Q.; Cheng, X. W.; Xu, Z. H.; Mou, J.; Mei, S. J.; Kong, D. F.; Wang, X.; Li, Y.; Fellmeth, G. and Ma, H. W.** (**2012**). *Estimate of 2009 H1N1 influenza cases in Shenzhen--the biggest migratory city in China.*, Epidemiol Infect 140 : 788-797.

**Xu, C.; Bai, T.; Iuliano, A. D.; Wang, M.; Yang, L.; Wen, L.; Zeng, Y.; Li, X.; Chen, T.; Wang, W.; Hu, Y.; Yang, L.; Li, Z.; Zou, S.; Li, D.; Wang, S.; Feng, Z.; Zhang, Y.; Yu, H.; Yang, W.; Wang, Y.; Widdowson, M.-A. and Shu, Y.** (**2011**). *The seroprevalence of pandemic influenza H1N1 (2009) virus in China.*, PLoS One 6 : e17919.

**Yang, F.; He, J.; Zhong, H.; Ke, C.; Zhang, X.; Hong, T.; Ni, H. and Lin, J.** (**2012d**). *Temporal trends of influenza A (H1N1) virus seroprevalence following 2009 pandemic wave in Guangdong, China: three cross-sectional serology surveys.*, PLoS One 7 : e38768.

**Yang, L.; Wang, X. L.; Chan, K. P.; Cao, P. H.; Lau, H. Y.; Peiris, J. S. and Wong, C. M.** (**2012a**). *Hospitalisation associated with the 2009 H1N1 pandemic and seasonal influenza in Hong Kong, 2005 to 2010.*, Euro Surveill 17.

**Yang, P.; Duan, W.; Lv, M.; Shi, W.; Peng, X.; Wang, X.; Lu, Y.; Liang, H.; Seale, H.; Pang, X. and Wang, Q.** (**2009**). *Review of an influenza surveillance system, Beijing, People's Republic of China.*, Emerg Infect Dis 15 : 1603-1608.

**Yang, P.; Qian, H.; Peng, X.; Liang, H.; Huang, F. and Wang, Q.** (**2010**). *Alternative epidemic of different types of influenza in 2009-2010 influenza season, China.*, Clin Infect Dis 51 : 631-632.

**Yang, P.; Shi, W.; Tian, L.; Li, S.; Zhang, L.; Huang, F. and Wang, Q.** (**2011**). *Serological surveillance of 2009 H1N1 influenza in China.*, Int J Infect Dis 15 : e151-e152.

**Yang, X.; Yao, Y.; Chen, M.; Yang, X.; Xie, Y.; Liu, Y.; Zhao, X.; Gao, Y. and Wei, L.** (**2012c**). *Etiology and clinical characteristics of influenza-like illness (ILI) in outpatients in Beijing, June 2010 to May 2011.*, PLoS One 7 : e28786.

**Yang, Z.; Dong, Z. and Fu, C.** (**2012e**). *Seasonal influenza vaccine effectiveness among children aged 6 to 59 months in southern China.*, PLoS One 7 : e30424.

**Yang, Z.-f.; Zhan, Y.-q.; Chen, R.-c.; Zhou, R.; Wang, Y.-t.; Luo, Y.; Jiang, M.; Li, J.-q.; Qin, S.; Guan, W.-d.; Lai, K.-f.; Wen, H.-l.; Liang, Z.-w.; Li, L. and Zhong, N.-s.** (**2012b**). *A prospective comparison of the epidemiological and clinical characteristics of pandemic (H1N1) 2009 influenza A virus and seasonal influenza A viruses in Guangzhou, South China in 2009.*, Jpn J Infect Dis 65 : 208-214.

**shan Yu, D.; yu Li, H. and ming Gao, X.** (**2010**). *[Analysis of influenza type A(H1N1) antibody levels in different population in Gansu province].*, Zhongguo Yi Miao He Mian Yi 16 : 219-21, 260.

**Yu, H.; Cauchemez, S.; Donnelly, C. A.; Zhou, L.; Feng, L.; Xiang, N.; Zheng, J.; Ye, M.; Huai, Y.; Liao, Q.; Peng, Z.; Feng, Y.; Jiang, H.; Yang, W.; Wang, Y.; Ferguson, N. M. and Feng, Z.** (**2012**). *Transmission dynamics, border entry screening, and school holidays during the 2009 influenza A (H1N1) pandemic, China.*, Emerg Infect Dis 18 : 758-766.

**Yu, H.; Feng, L.; Peng, Z.; Feng, Z.; Shay, D. K. and Yang, W.** (**2009**). *Estimates of the impact of a future influenza pandemic in China.*, Influenza Other Respi Viruses 3 : 223-231.

**Zheng, Y.; Duan, W.; Yang, P.; Zhang, Y.; Wang, X.; Zhang, L.; Liyanage, S. S. and Wang, Q.** (**2013**). *Risk Factors for influenza A(H1N1)pdm09 among students, Beijing, China.*, Emerg Infect Dis 19 : 309-312.

**Zhou, Y.; Ng, D. M. W.; Seto, W.-H.; Ip, D. K. M.; Kwok, H. K. H.; Ma, E. S. K.; Ng, S.; Lau, L. L. H.; Peiris, J. S. M. and Cowling, B. J.** (**2011**). *Seroprevalence of pandemic H1N1 antibody among health care workers in Hong Kong following receipt of monovalent 2009 H1N1 influenza vaccine.*, PLoS One 6 : e27169.

**Zhu, M.; Zu, R.-q.; Huo, X.; Bao, C.-j.; Zhao, Y.; Peng, Z.-h.; Yu, R.-b.; Shen, H.-b. and Chen, F.** (**2011**). *[The application of time series analysis in predicting the influenza incidence and early warning].*, Zhonghua Yu Fang Yi Xue Za Zhi 45 : 1108-1111.
